# Mucosal-Associated Invariant T cells exhibit distinct functional signatures associated with protection against typhoid fever

**DOI:** 10.1016/j.cellimm.2022.104572

**Published:** 2022-06-20

**Authors:** Rosângela Salerno-Gonçalves, Stephanie Fresnay, Laurence Magder, Thomas C. Darton, Claire S. Waddington, Christoph J. Blohmke, Brian Angus, Myron M. Levine, Andrew J. Pollard, Marcelo B. Sztein

**Affiliations:** a Center for Vaccine Development & Global Health, University of Maryland School of Medicine, Baltimore, MD, USA; b Department of Epidemiology and Public Health, University of Maryland School of Medicine, Baltimore, MD, USA; c Oxford Vaccine Group, Department of Paediatrics, University of Oxford, and the NIHR Oxford Biomedical Research Centre, Oxford, UK

**Keywords:** MAIT cells, Human, Polyfunctionality, Disease status, Bacteria, *Salmonella*

## Abstract

We have previously demonstrated that Mucosal-Associated Invariant T (MAIT) cells secrete multiple cytokines after exposure to *Salmonella enterica* serovar Typhi (*S*. Typhi), the causative agent of typhoid fever in humans. However, whether cytokine secreting MAIT cells can enhance or attenuate the clinical severity of bacterial infections remain debatable. This study characterizes human MAIT cell functions in subjects participating in a wild-type *S*. Typhi human challenge model. Here, we found that MAIT cells exhibit distinct functional signatures associated with protection against typhoid fever. We also observed that the cytokine patterns of MAIT cell responses, rather than the average number of cytokines expressed, are more predictive of typhoid fever outcomes. These results might enable us to objectively, based on functional parameters, identify cytokine patterns that may serve as predictive biomarkers during natural infection and vaccination.

## 1. Introduction

Our recent observations support the idea that polyfunctional T cells might contribute to protection against typhoid fever (TF) [1–6]. CD8 + MAIT cells, which make up a subset of T cells, are also likely to play a role in the development of typhoid fever [7]. Using samples of a wild-type *S*. Typhi human challenge model, we found that in contrast to participants who did not meet the clinical typhoid fever definition, participants who did exhibit a sharp decline of circulating CD8 + MAIT cells after diagnosis, in the timeframe immediately after the onset of clinical typhoid fever [7]. We also observed that after *in vitro S*. Typhi exposure, CD8 + MAIT cells can acquire multiple profiles of IFN-γ, TNF-α and IL-17A cytokine secretion [8]. However, in contrast to conventional T cells, there are no published data on the impact of MAIT cell polyfunctionality on the progression of typhoid fever. In fact, there is a debate on whether cytokine secreting MAIT cells can enhance or attenuate the clinical severity of bacterial infections [9–11]. Emgard and colleagues showed that MAIT cells are the primary source of IFN-γ and TNF-β in the early stages of the immunological response to group A streptococci, likely contributing to the pathological cytokine storm underlying Streptococcal toxic shock syndrome [12]. In contrast, Grimaldi *et al*. found that patients with severe bacterial infections in intensive care units (ICU) displayed early decreases in MAIT cell counts in blood compared with healthy control subjects [13]. Of interest, in a murine sepsis model, MAIT cell-deficient mice had significantly reduced levels of lung IFN-γ, TNF-α, IL-17A, IL-10, and GM-CSF cytokines compared with wild-type mice [14]. In another study using murine respiratory tularemia as a model of mucosal infection, Meierovics *et al*. found that in the absence of MAIT cells, mice had a defect in the production of IFN-γ, IL-17A, and TNF-α that precluded control of *Francisella tularensis* infection [15]. Thus, pursuing a longitudinal *ex vivo* analysis of MAIT cell functions after wild-type *S*. Typhi infection may help understand the dynamics of these responses.

In the present study, we characterized human MAIT cell function in volunteers participating in a wild-type *S*. Typhi human challenge model [7,16]. Challenge models offer enormous potential to study disease pathogenesis and accelerate vaccine development, especially regarding human-restricted pathogens like *S*. Typhi [17]. Drs. Theodore Woodward and Richard B. Hornick at the University of Maryland in Baltimore established the typhoid challenge model, extensively used from 1952 to 1974 [18,19], which led to a better understanding of typhoid fever pathogenesis and facilitated the development of the live attenuated typhoid vaccine Ty21a [20]. Dr. Pollard’s Oxford Vaccine Group initiated experimental challenge studies of community participants using the *S*. Typhi strain Quailes previously used in many challenge trials in Maryland [16]. Specimens from the first Oxford challenge study were used to generate the data in this manuscript [16]. Twenty participants were divided into two groups: TF (i.e., participants who had fever ≥ 38 °C followed by *S*. Typhi isolation from blood) and NoTF (i.e., participants who did not meet the typhoid fever definition). The NoTF and TF definitions were used to designate a “case” of typhoid fever and are similar to the ones used in the Maryland challenge model and surveillance in field trials to measure vaccine efficacy. We found that MAIT cells isolated from NoTF and TF exhibited distinct functional signatures. We also observed that the cytokine patterns of MAIT cell responses, rather than the average number of cytokines expressed, is more predictive of disease outcome.

## 2. Material and methods

### 2.1. Ethics statement

The protocol for blood collection in the wild-type *S*. Typhi challenge model was approved by National Research Ethics Seivice (NRES), Oxfordshire Research Ethics Committee A (10/H0604/53). The clinical trial was conducted under the ethical standards laid down in the 1964 Declaration of Helsinki and the principles of the International Conference on Harmonization Good Clinical Practice guidelines [16,21]. Participants were informed about the research objectives and gave informed, signed consent before the blood draws. All blood specimens were processed within 4 h of obtaining the blood specimens.

### 2.2. Subjects

Healthy participants between 19 and 46 years old were screened for good health by medical history, physical examination, and normal laboratory tests, as previously described [7,16]. Briefly, participants who had not previously received a typhoid vaccination and who had not resided in typhoid-endemic areas for > 6 months were enrolled in this study. These participants received a dose of the inoculum, the antibiotic susceptible, virulent wild-type *S*. Typhi strain Quailes. Twenty participants whose peripheral blood mononuclear cells (PBMC) were available were divided into two groups: 13 participants who did not meet the clinical typhoid fever definition (NoTF) and 7 who did (TF) (S1 Table). Determination of typhoid fever was based on a fever ≥ 38 ° C followed by positive blood culture. This clinical typhoid fever definition is similar to the endpoint used in the Maryland challenge model and to surveillance in field trials to measure vaccine efficacy [17,20,22]. PBMC were isolated from blood by density gradient centrifugation and cryopreserved in liquid N_2_ following standard techniques [16]. PBMC collected before and up to 28 days after the challenge were evaluated in the studies included in this manuscript.

### 2.3. Antibodies and cell culture media

Cells were stained with anti-human monoclonal antibodies (mAbs) to CD3 (clone OKT3), CD14 (clone M5E2), CD19 (clone HIB19), CD45 (clone HI30), CD161 (clone HP-3G10), TCR Vα7.2 (clone 3C10) (Biolegend, San Diego, CA), CD4 (clone L200), CD8 (clone SK1), CCR6 (clone 11A9), IFN-γ (clone B27), TNF-α (clone MAb11) (BD Pharmingen, San Diego, CA), CCR9 (clone 112509; R&D, Minneapolis, MN, USA), CD69 (clone TPI-55–3) (Beckman-Coulter, Miami, FL), CD57 (clone TB01), and IL-17A (clone eBio64DEC17) (eBioscience, San Diego, CA). Antibodies conjugated to the following fluorochromes were used in these studies: Fluorescein isothiocyanate (FITC), Phycoerythrin (PE), Peridinin chlorophyll protein (PerCP)-Cy5.5, PE-Cy7, Energy Coupled Dye or PE-Texas-Red conjugate (ECD), Violet (V) 450 (e.g., similar to Pacific Blue), Brilliant Violet (BV) 570, BV605, BV650, Quantum dot (QD) 800, Alexa 647, allophycocyanin (APC)-Alexa 700 and APC-H7.

The culture medium consisted of RPMI 1640 (Gibco, Grand Island, New York) supplemented with 100 U/ml penicillin, 100 μg/ml streptomycin, 50 μg/ml gentamicin, 2 mM l-glutamine, 2.5 mM sodium pyruvate, 10 mM HEPES buffer, and 10% heat-inactivated fetal bovine serum (R10).

### 2.4. S. Typhi infection and co-culture of MAIT cells with target cells

B-LCLs were used as target cells, and generated, cultured, and infected with *S*. Typhi as previously described [23]. Briefly, autologous B-LCLs were exposed to wild-type *S*. Typhi strain Ty2 at 1:3 cell:bacteria multiplicity of infection (MOI)[6] for 3 h. Uninfected cells were used as controls. After 16–18 h of gentamicin treatment, B-LCLs were irradiated (6,000 rads) and surface stained with a mAb to CD45, a marker abundantly expressed on the surface of hematopoietic cells [24]. After staining, autologous B-LCL were co-cultured with overnight-rested PBMC at a B-LCL:PBMC cell ratio of 1:5 in R10 media for an additional 16–18 h at 37 °C, 5% CO_2_.

### 2.5. Surface and intracellular staining

After 16–18 h of co-culture, cells were harvested, stained with a dead-cell discriminator, yellow fluorescent viability dye (YEVID, Invitrogen, Carlsbad, CA) [25], followed by surface staining with mAbs to CCR6, CCR9, CD3, CD4, CD8, CD14, CD19, CD57, CD161, and TCRα 7.2, and fixation and permeabilization with Fix & Perm cell buffers (Invitrogen, Carlsbad, CA)[8,25]. Cells were then stained intracellularly for CD69, IL-17A, IFN-γ, and TNF-α. Finally, cells were resuspended in fixation buffer (1% formaldehyde) and analyzed by flow cytometry on an LSR-II instrument (BD Biosciences). Data were analyzed with WinList v9.0.1 (Verity Software House, Topsham, ME). Lymphocytes were gated based on their light scatter characteristics. Single lymphocytes were gated based on forward scatter height vs. forward scatter area. A dump channel was used to exclude dead cells (YEVID^+^), macrophages/monocytes (CD14^+^), and B lymphocytes (CD19^+^) from the analysis. This step was followed by additional gating on CD3, CD4, CD8, CD161, and TCR Vα7.2 to identify CD8 + MAIT cells. To measure specific MAIT cell responses to *in vitro* stimulation by autologous B-LCL, cytokine secreting MAIT cells were gated based on cytokine production in cells expressing the early activation marker CD69. To ensure sufficient MAIT cell events would be available for analysis, we routinely collected 300,000–500,000 events in the forward and side scatter (FS/SS) lymphocyte gate during sample acquisition.

### 2.6. Statistical analyses

The statistical analyses were performed using SAS 9.3 (Cary, NC) or Prism software (version 7, GraphPad Software, La Jolla, CA). A two-sided paired Student’s *t*-test was used for comparisons among two groups. Mixed-effects models were used to compare mean values by time period and group while accounting for correlations between multiple measures from the same participant at the same time period and across time periods. These models, which include a random effect for the subject, were fit by restricted maximum likelihood. Receiver operating characteristic (ROC) area under the curve (AUC) analyses were performed to assess the cytokine level’s capability to discriminate between NoTF and TF volunteers. Discrimination is assumed to be effective if AUC ≥ 0.75. The AUC scores are: 0.9–1.0 excellent, 0.8–0.9 good, 0.7–0.8 fair, and 0.6–0.7 poor discrimination performance [26]. Where indicated, observations were grouped in the following periods: pre-challenge (day 0), days 1 to 4, days 7 to 10 or within 48–96 h of disease onset, and days 14–28. *P* values < 0.05 were considered significant. To visualize the variance of the 7 possible FCOM *T*-cell phenotypes, we performed Principal Component Analysis (PCA) as described previously with small modifications [27,28]. Briefly, the calculation of principal components was performed by ClustVis web tools [29]. Unit variance scaling was applied to rows; singular value decomposition (SVD) with imputation was used to calculate principal components.

## 3. Results

### 3.1. MAIT cell function after experimental challenge with wild-type S. Typhi in humans

**Fig. 1.**
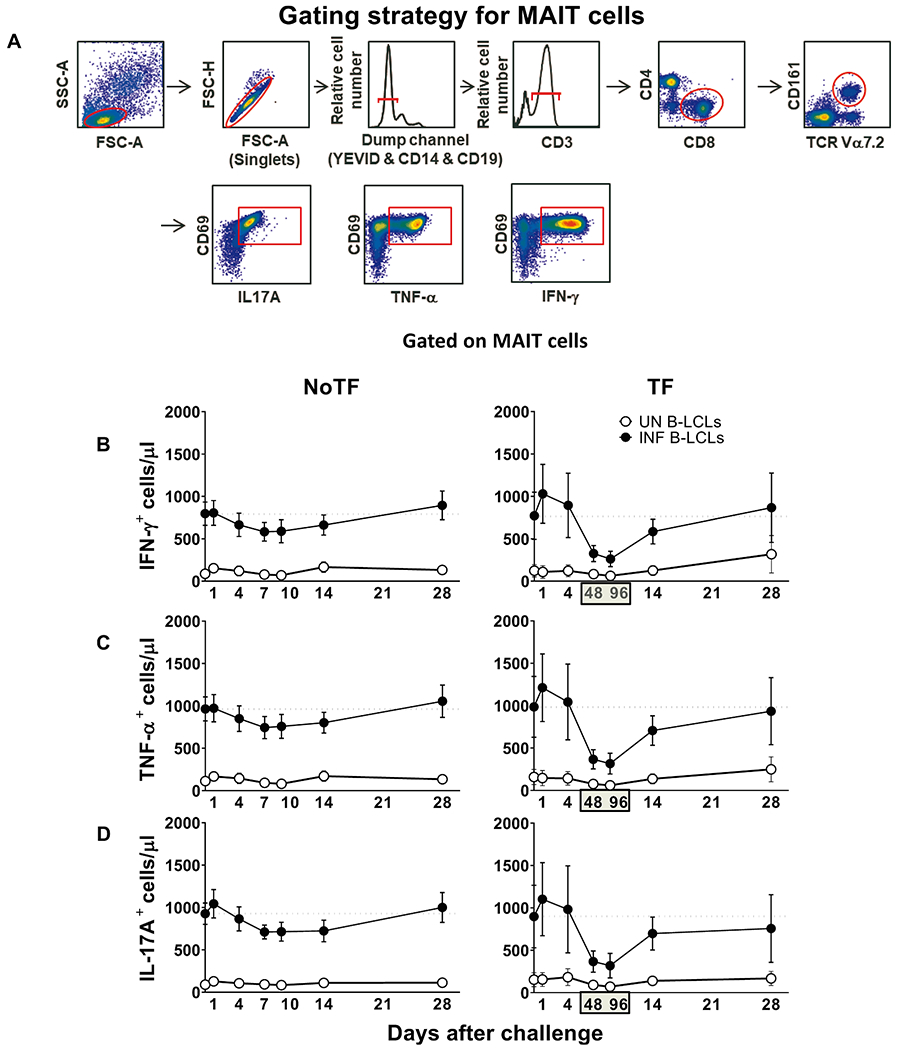
Kinetics of cytokine-producing MAIT cells from participants over a 28-day post-challenge follow-up period. *Ex vivo* PBMC from participants receiving a *S*. Typhi inoculum were stained with YEVID, followed by surface staining with mAbs to CD3, CD4, CD8, CD14, CD19, CD161, and TCRα 7.2. After fixation and permeabilization, cells were intracellularly stained with monoclonal antibodies to CD69, as well as to IFN-γ, TNF-αα, and IL-17A cytokines and analyzed by flow cytometry. For the analysis, following the elimination of doublets and other debris, a “dump” channel was used to eliminate dead cells (YEVID^+^) as well as macrophages (CD14^+^), and B cells (CD19^+^) from the analyses. This was followed by additional gating on CD3, CD4, and CD8, as well as CD161 versus TCRα 7.2 to analyze MAIT cells, and afterward on CD69, IFN-γ, TNF-α, and IL-17A to evaluate cytokine secretion. (**A**) Representative gating strategy for MAIT cells. Kinetics of the production of (**B**) IFN-γ, (**C**) TNF-α, and (**D**) IL-17A by MAIT cells following exposure to (●) autologous B-LCLs infected with *S*. Typhi (INF B-LCLs), or controls (○) (UN, uninfected B-LCLs). The curves represent the mean, and the error bars denote the standard errors of the results from the 13 participants who did not meet the clinical typhoid fever definition (NoTF), and the 7 who did (TF). The dashed lines represent the baseline values (day 0). Numbers in the “X” axis represent days after challenge, except for the numbers inside of the green box that represent 48 and 96 hrs after diagnosis of typhoid disease. Data are presented as absolute MAIT cell numbers per microliter of peripheral blood.

**Fig. 2.**
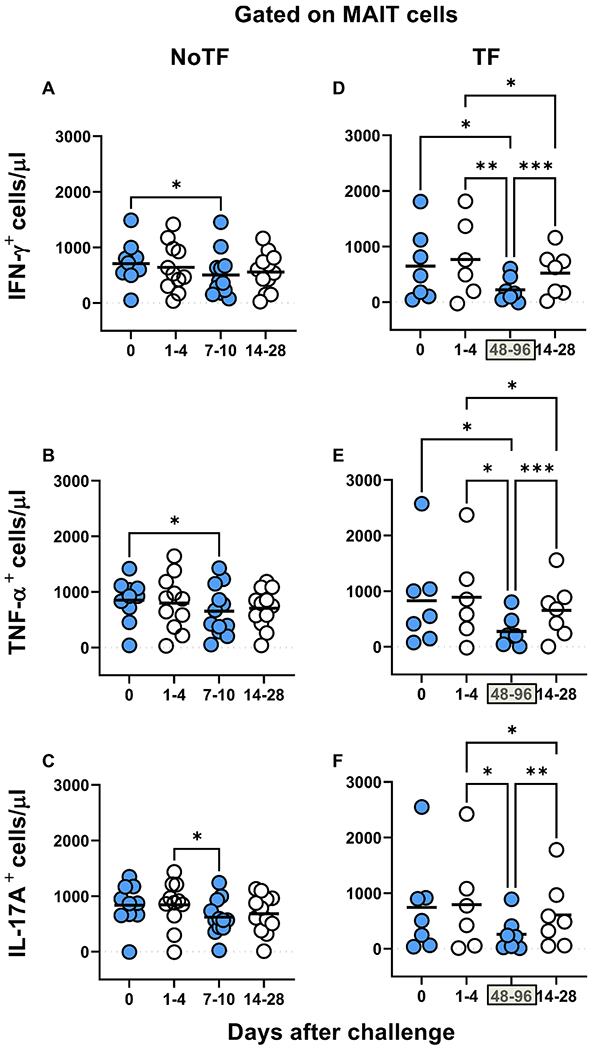
Comparison of the cytokine-producing MAIT cell kinetics over the 28-day post-challenge follow-up period. *Ex vivo* PBMC from participants receiving the *S*. Typhi inoculum were stained and analyzed as described in the legend to Fig. 1. Levels of MAIT cells expressing IFN-γ(**A** & **D**), TNF-α(**B** & **E**), and IL-17A (**C** & **F**), were evaluated in NoTF and TF participants. Data were grouped by time frames as follows: day 0, days 1–4, days 7–10 (for NoTF), or 48–96 h for TF participants and days 14–28. For grouped timepoints, each dot represents the mean value of the different timepoints for a specific participant. NoTF, participants who did not meet the clinical typhoid fever definition; TF, participants who did meet the clinical typhoid fever definition. *, represent significant differences (*P* < 0.05). Data are presented as absolute MAIT cell numbers per microliter of peripheral blood.

Our previous work showed that MAIT cells exposed *in vitro* to *S*. Typhi-infected cells secrete multiple cytokines, including IL-17A, IFN-γ, and TNF-α [8]. However, it is not clear whether cytokine secreting MAIT cells can enhance or attenuate the clinical severity of bacterial infections. Thus, pursuing a longitudinal analysis of MAIT cell functions, before and after wild-type *S*. Typhi infection, will give us a unique opportunity to understand better the contribution of the cytokines produced by MAIT cells to clinical outcomes. To this end, *ex vivo* PBMC, collected before and up to 28 days after challenge with wild-type *S*. Typhi, were stimulated with *S*. Typhi-infected autologous B-lymphoblastoid cell lines (B-LCLs) and IL-17A, IFN-γ and TNF-α cytokine production measured by flow cytometry. PBMC from 20 participants were divided into 2 groups: 13 participants who did not meet the clinical typhoid fever definition (NoTF) and 7 participants who developed typhoid fever (TF; fever > 38 °C plus *S*. Typhi isolated from blood in a culture obtained after the onset of fever) (S1 Table). We determined the percentages and absolute numbers of cytokine secreting MAIT cells. To this end, absolute MAIT cell numbers were calculated by multiplying their percentages as measured by flow cytometry by the lymphocyte numbers in whole blood (per microliter) and dividing it by 100. Regardless of the disease status, following exposure to *S*. Typhi-infected autologous B-LCLs, we observed an increase in the percentages and absolute numbers of MAIT cells secreting either IFN-γ, TNF-α or IL-17A (Fig. 1 & S1 Figure) compared to MAIT cells exposed to uninfected B-LCLs. Of note, the levels of cytokine-secreting MAIT cells varied among participants (S2 & S3 Figures). Consistent with our previous study [7], MAIT cell responses were then grouped by the days subsequent challenge in the following periods: pre-challenge (day 0), days 1 to 4, days 7 to 10 or within 48–96 h of disease onset, and days 14–28. Regardless of the disease status, and except for IL-17A in TF, following exposure to autologous *S*. Typhi-infected B-LCLs, we observed a sharp decline in the counts of cytokine secreting MAIT cells on days 7–10 (48–96 hs) compared to pre-challenge (day 0). However, these declines were greater in the TF group. In TF participants, we also observed significant decreases in the levels of cytokine-secreting MAIT cells 48–96 hs after typhoid diagnosis compared to days 1–4 and 14–28 (Fig. 2D–F). Interestingly, these declines were not apparent or did not reach significance when using percentages as an outcome measure to evaluate MAIT cell cytokine patterns over time (S4 Figure). Finally, although the counts of MAIT cells secreting TNF-αα and IL-17A were significantly lower in TF than in NoTF participants at 48–96 hs (days 7–10 for NoTF) (S5A Figure), no significant differences, just trends, were observed between these two groups when using MAIT cell percentages as an outcome measure to evaluate their patterns of cytokine production across timepoints (S5B Figure).

### 3.2. Cumulative MAIT cell responses over the 28-day follow-up period

To circumvent the relatively small number of volunteers in our study, their variability in cytokine production, and to increase the power to find patterns associated with disease progression, we performed cumulative measurements of the level and duration of cytokine production by MAIT cells as a single parameter. We used Receiver Operating Characteristic (ROC) Area Under the Curve (AUC) (ROC AUC) analysis to discriminate NoTF and TF based on the production of cytokines by MAIT cells. The ROC AUC is a measure of the true positive rates (Sensitivity) in function of false-positive rates (100-Specificity) [30,31]. Its predicted values vary from 0 to 1, where the higher its predictive value, the better the accuracy [26,32]. As shown in S6 Figure, the ROC AUC showed no discriminative ability of total IFN-γ (AUC = 0.597), and TNF-α (AUC = 0.5972), but a good discriminative ability of IL-17A (AUC = 0.875; *P* = 0.0114). This suggests that the functional signature of IL-17A secreting MAIT cells might predict disease outcomes.

### 3.3. Induction of polyfunctional MAIT after S. Typhi exposure

**Fig. 3.**
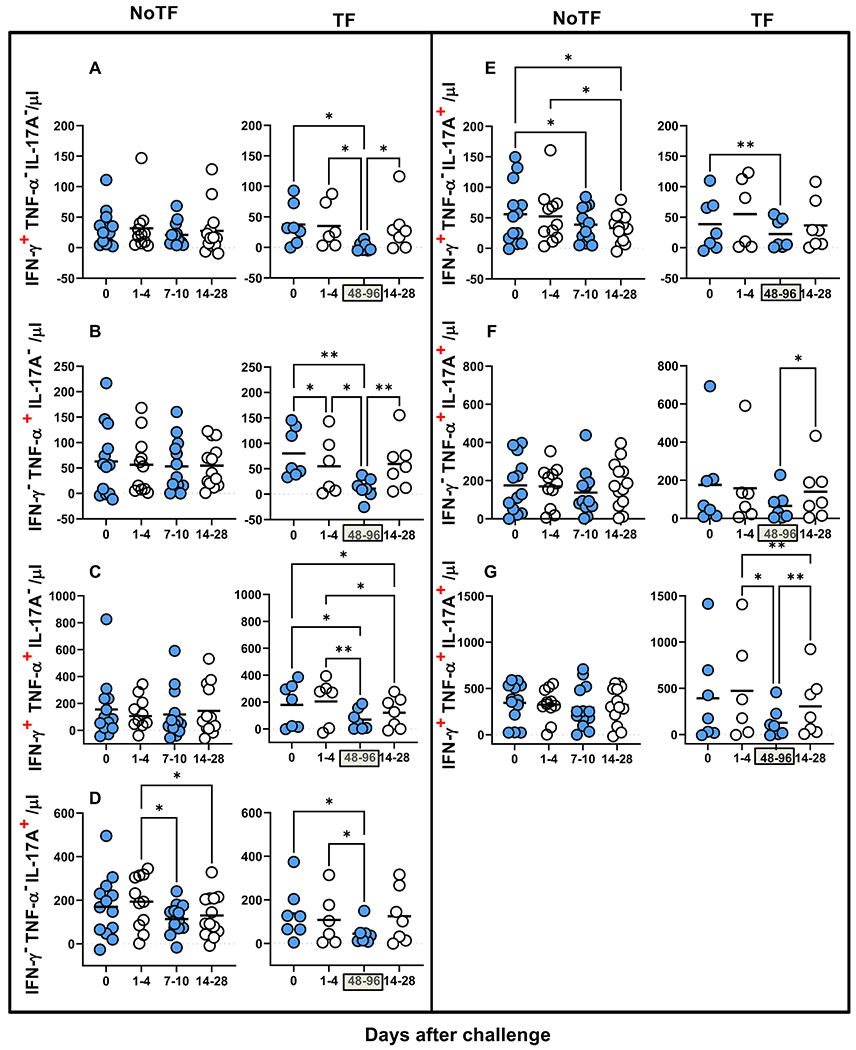
Comparison of mono and polyfunctional MAIT cell kinetics over a 28-day post-challenge follow-up period. *Ex vivo* PBMC from participants receiving the *S*. Typhi inoculum were stained and MAIT cells gated as described in Fig. 1. FCOM, an analysis tool that automatically reduces multiparameter data to a series of multiple event acquisition gates, one for every possible sub-phenotype, was employed to study MAIT cell polyfunctionality. Based on the pre-defined positive staining regions for cytokines, FCOM calculated 7 possible phenotypes as displayed in the figure legend. Data are representative of 13 participants who did not meet the clinical typhoid fever definition (NoTF), and 7 who did (TF). Data are the net responses calculated by subtracting the MAIT cell responses of the controls (uninfected B-LCLs) from those observed to B-LCLs infected with *S*. Typhi, and grouped by time frames as follows: day 0, days 1–4, days 7–10 (for NoTF, or 48–96 h for TF participants), and days 14–28. For grouped timepoints, each dot represents the mean value of the different timepoints for a specific participant. Asterisks describe the levels of statistical significance as: **, very significant (*P*-value between 0.001 and 0.01); *, significant (*P*-value between 0.01 and 0.049); *P* values < 0.05 were considered statistically significant. Data are presented as absolute MAIT cell numbers per micro liter of peripheral blood expressing a particular cytokine combination.

**Fig. 4.**
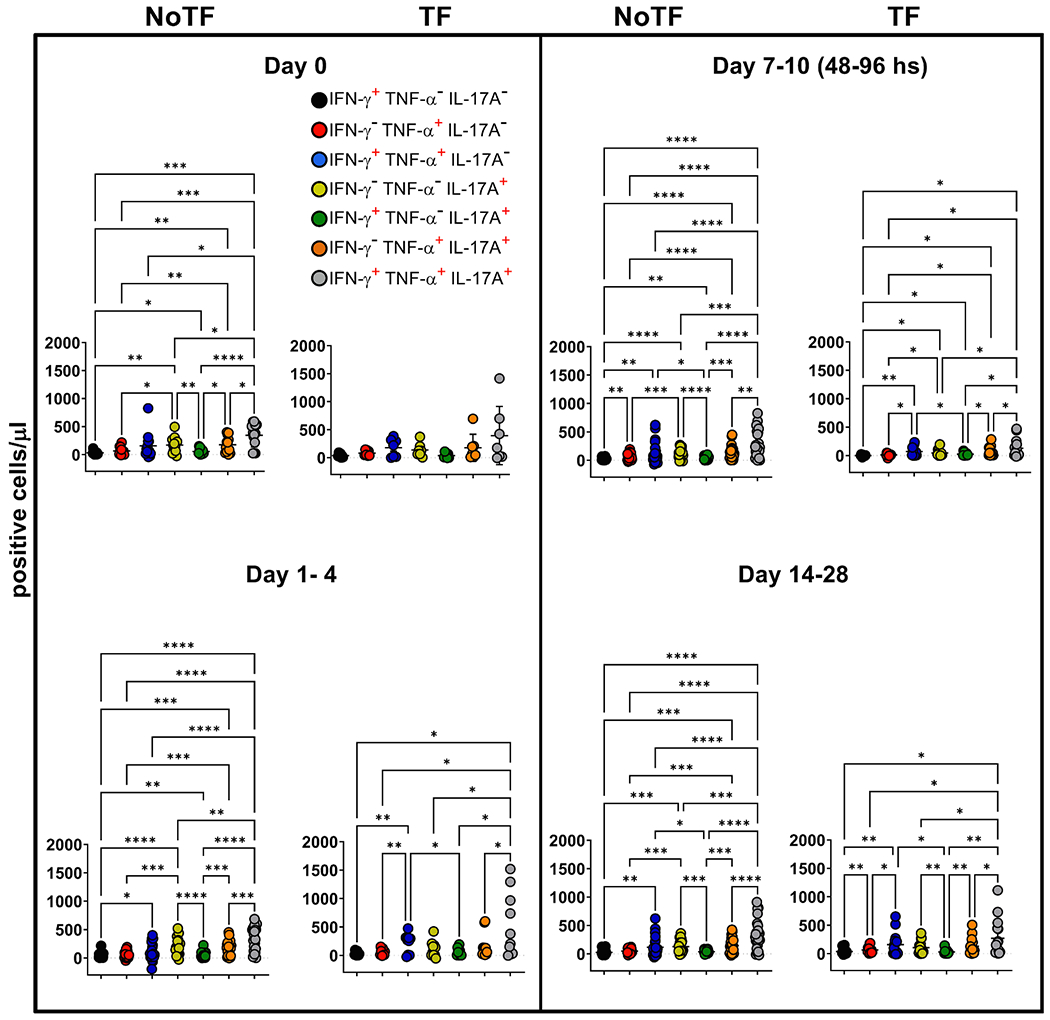
Comparison among monofunctional and polyfunctional MAIT cells over a 28-day post-challenge follow-up period. *Ex vivo* PBMC were stained and MAIT cells gated as described in Fig. 1. FCOM, an analysis tool that automatically reduces multiparameter data to a series of multiple event acquisition gates, one for every possible sub-phenotype, was employed to study MAIT cell polyfunctionality. Based on the pre-defined positive staining regions for cytokines, FCOM calculated 7 possible phenotypes as displayed in the figure legend. Data are representative of 13 participants who did not meet the clinical typhoid fever definition (NoTF), and 7 who did (TF). Data are the net responses calculated by subtracting the MAIT cell responses of the controls (uninfected B-LCLs) from those observed to B-LCLs infected with *S*. Typhi, and grouped by time frames as follows: day 0, days 1–4, days 7–10 (for NoTF, or 48–96 h for TF participants) and days 14–28. Asterisks describe the levels of statistical significance as: ****, extremely significant (*P* < 0.0001); ***, extremely significant (*P*-value between 0.0001 and 0.001); **, very significant (*P*-value between 0.001 and 0.01); *, significant (*P*-value between 0.01 and 0.049); *P* values < 0.05 were considered statistically significant. Data are presented as absolute MAIT cell numbers per microliter of peripheral blood expressing a particular cytokine combination.

Previously we showed that after *S*. Typhi exposure, CD8 + MAIT cells became polyfunctional and produced multiple profiles of IFN-γ, TNF-α, and IL-17A cytokines [8]. Thus, we concurrently measured MAIT cell production of IFN-γ, TNF-α, and IL-17A by multichromatic flow cytometry using the FCOM tool of the WinList software. FCOM allows us to evaluate all 7 possible cytokine combinations and quantify each cytokine producing MAIT cell subset. When comparing the magnitude of NoTF responses over time, except for IFN-γ^−^ TNF-α^−^ IL-17A^+^ and IFN-γ^+^ TNF-α^−^ IL-17A^+^ MAIT subsets on days 7–10 and 14–28 (Fig. 3D & E), only minimal perturbations of the absolute counts of the other MAIT subsets were observed across time points (Fig. 3A–C & F–G). In contrast, we observed significant decreases in the absolute counts of all cytokine secreting MAIT cell subsets in TF participants after typhoid diagnosis (48–96 hs) (Fig. 3A–G). In addition, as it was the case for total cytokines, measures of the percentages of cytokine secreting MAIT cell subsets were generally less sensitive to capture the pattern changes across timepoints (S7 Figure). It is important to note that regardless of the disease status, most MAIT cell responses were characterized by triple cytokine production (IFN-γ^+^TNF-α^+^IL-17A^+^) compared to single and double-positive cytokine secreting MAIT cells (Fig. 4 & S8 Figure). In agreement with our previous work, IL-17A production in combination with other cytokines was mainly linked to the presence of TNF-α (Fig. 4 & S8 Figure). MAIT cells that produce concomitantly IFN-γ and IL-17A but not TNF-α were present at a very low frequency (Fig. 4 & S8 Figure). MAIT cells that produced only IFN-γ and but not IL-17A or TNF-α were also present at very low frequencies (Fig. 4 & S8 Figure). Interestingly, NoTF exhibited a sizable increase of MAIT cells that produce only IFN-γ (S9A Figure), TNF-α (S9E Figure), or IL-17A (S9F Figure) at days 7–10 compared to TF at 48–96 hs. Once again, measures of cytokine secreting MAIT cell percentages were generally less sensitive to capture the changes in the patterns of cytokine production over time, although trends were present (S10 Figure). These results further confirm that the quality of MAIT cell responses varies in function of disease outcome. Finally, since the counts of MAIT cells appeared to be a more reliable measure than their percentages as an outcome measure to observe changes in the quality of their responses, henceforward, all analyses were performed by normalizing the levels of MAIT cells based on the absolute numbers of lymphocytes.

### 3.4. MAIT cell functional signatures and their association with disease status

**Fig. 5.**
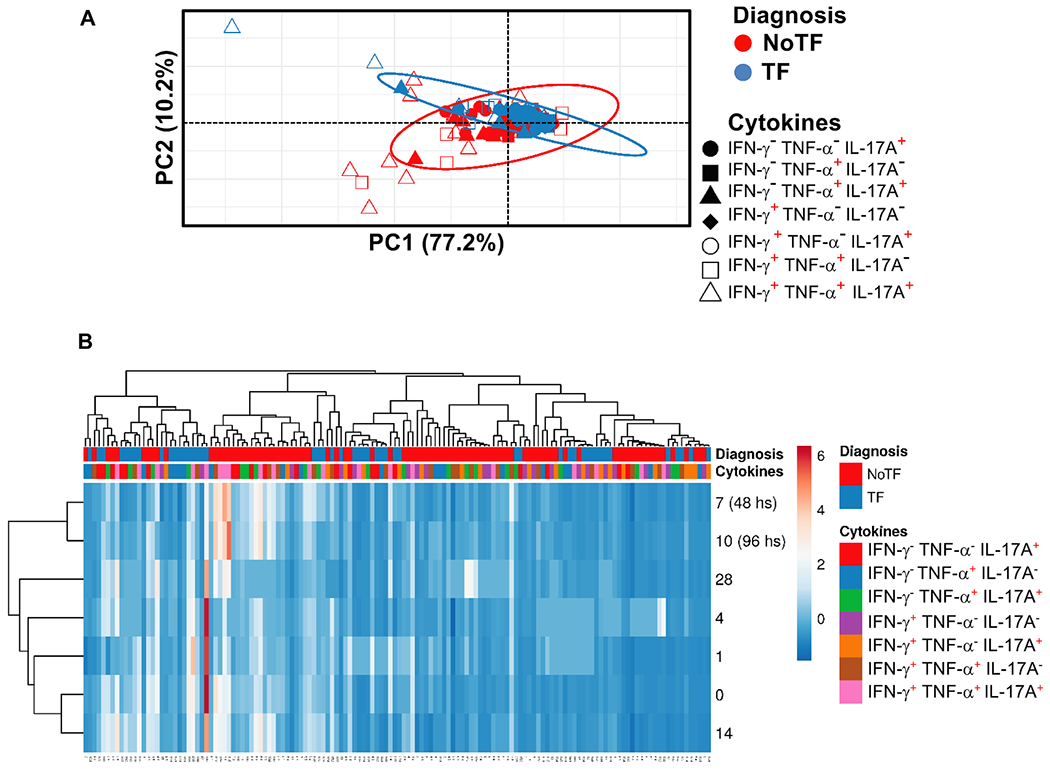
Hierarchical clustering of MAIT cell functions using principal component analysis (PCA). The three MAIT cell functions (IFN-γ, TNF-α, and IL-17A production) were analyzed using the FCOM deconvolution tool. FCOM data of the 7 possible combinations were used to perform an unsupervised PCA analysis. All data from NoTF and TF participants were merged for combined analysis, generating a matrix of the 7 possible phenotypes of cytokine producing MAIT cell subsets from the 20 participants at 7 different time points. (**A**) PCA. X and Y axis show principal component 1 and principal component 2, which account for 77.2% and 10.2% of the total variance, respectively. Prediction ellipses are such that with a probability of 0.95, a new observation from the same group will fall inside the ellipse (N = 140 data points). (**B**) Heatmap. Rows are centered; unit variance scaling was applied to rows. Imputation was used for missing value estimation. Both rows and columns are clustered using correlation distance and average linkage. 7 rows, 140 columns. PCA was generated using absolute MAIT cell numbers per microliter of peripheral blood.

**Fig. 6.**
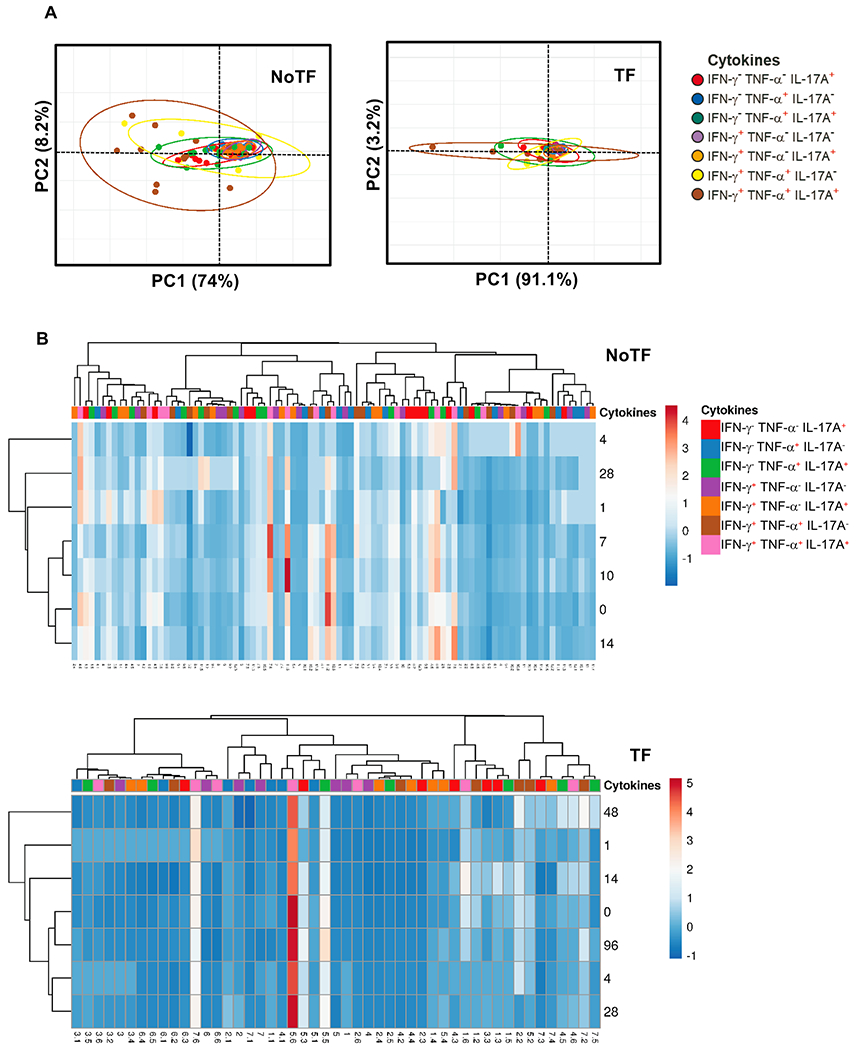
PCA plots for NoTF and TF clustering. The three MAIT cell functions (IFN-γ, TNF-α, and IL-17A production) were analyzed using the FCOM deconvolution tool. FCOM data of the 7 possible combinations were used to perform an unsupervised PCA analysis. Data from NoTF (91 data points) and TF (49 data points) were processed separately. Unit variance scaling was applied to rows; SVD with imputation was used to calculate principal components. X and Y axis show principal component 1 and principal component 2. Prediction ellipses are such that with a probability of 0.95, a new observation from the same group will fall inside the ellipse. (**A**) PCA and (**B**) Heatmap. Rows are centered; unit variance scaling was applied to rows. Imputation was used for missing value estimation. Both rows and columns are clustered using correlation distance and average linkage. PCA and heatmap were generated using absolute MAIT cell numbers per microliter of peripheral blood.

We next evaluated the MAIT cell functional signatures using unsupervised Principal Component Analysis (PCA). To increase the statistical power, all data from NoTF and TF were merged for the combined analysis, generating a matrix of the 7 possible phenotypes of cytokine producing MAIT cell subsets from the 20 participants at 7 different time points (N = 140 data points). Since the absolute lymphocyte values in peripheral blood were missing for all participants on day 2, and most participants on day 21, these two time points were eliminated from the analysis. PCA analysis revealed that the first principal component (PC1) accounted for most of the total variance (77.2%) (Fig. 5A). Analysis of PC1 vs. PC2 loadings showed that TF MAIT T cell subsets displayed tighter clustering than NoTF MAIT cell subsets (Fig. 5A). This finding indicates that, in most cases, TF MAIT cell subsets are positively correlated, suggesting that all subsets behave in unison. When the numeral value of one subset increases or decreases, the numeral value of the other subsets tends to change in the same direction, contributing evenly to the variances. It is important to note that the IFN-γ^+^TNF-α^−^IL-17A^−^ (○) and IFN-γ^+^TNF-α^−^ IL-17A^+^(◆) subsets did not appear to be associated with disease status since their data completely overlapped between NoTF and TF MAIT cell subsets (Fig. 5A). Given the differences in cluster tightness among NoTF and TF MAIT cell subsets, we speculated that NoTF and TF MAIT cell subsets might behave differently. To explore this possibility, we deconvoluted the data and re-run the NoTF and TF PCA analyses separately (Fig. 6A). We observed that the PCA data clustering of IFN-γ^−^ TNF-α^−^ IL-17A^+^ (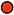), IFN-γ^−^ TNF-α^+^IL-17A^+^(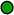), and IFN-γ^+^ TNF-α^+^ IL-17A^−^ (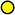) MAIT cell subsets from NoTF are looser than their counterparts in TF participants, suggesting differences in their contributions to the disease outcome (Fig. 6A). Interestingly, IFN-γ^+^TNF-α^+^IL-17A^+^ (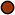) MAIT cell subset tends to score higher on the PCA for both NoTF and TF participants and might therefore not be a variant associated with disease outcome (Fig. 6A). We next plotted NoTF and TF combined data as a dendrogram diagram to evaluate the hierarchical relationship between MAIT cell subsets at various time points after exposure to *S*. Typhi (Fig. 5B). We found a connection between the variances on days 7–10/48–96 hs and the observations at other time points after the challenge (Fig. 5B). However, when we deconvoluted the data and re-run the NoTF and TF dendrogram diagram analyses separately, we found distinct patterns for MAIT cells from NoTF and TF individuals. While in NoTF, the dendrogram arrangement suggested a connection between the variances of MAIT cell subsets on day 4 and those at later time points after the challenge (*i.e.*, days 7–10/48–96 hs) (Fig. 6B), in TF, the dendrogram arrangement suggested a connection between the variances of MAIT cell subsets 48 hs after diagnosis and the observations at other time points after the challenge (Fig. 6B). These results confirm the PCA findings showing that cytokine secreting MAIT cell subsets from NoTF and TF behave differently.

### 3.5. Cumulative polyfunctional MAIT cell responses and their predictive values to discriminate NoTF and TF volunteers

**Fig. 7.**
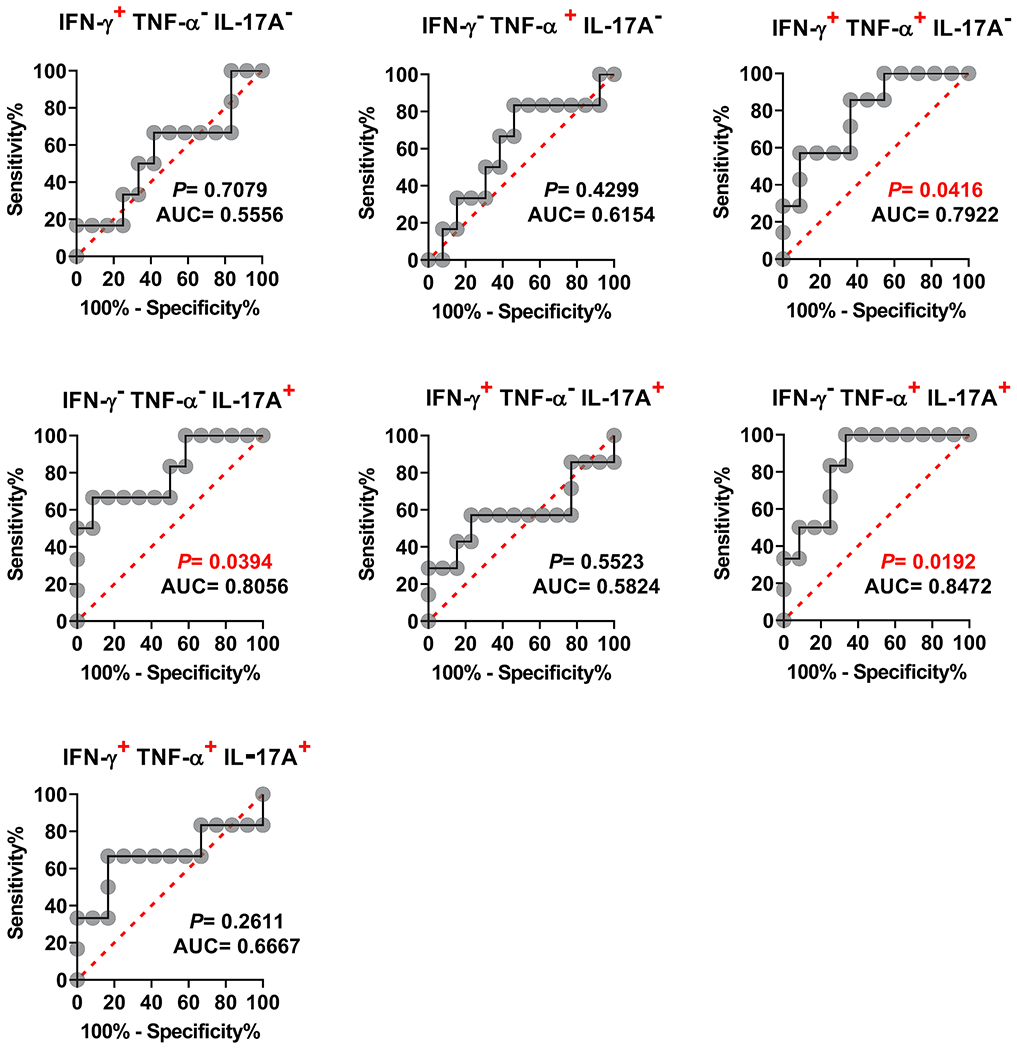
Ability of ROC AUC curves of cytokine secreting MAIT cells to discriminate clinical outcome in volunteers receiving wild-type *S*. Typhi. *Ex vivo* PBMC were stained, and IFN-γ, TNF-α, and IL-17A secreting MAIT cells gated as described in Fig. 1. ROC curve logistic regressions were based on net MAIT cell responses of volunteers who did not meet the definition of typhoid fever (NoTF), and those who did (TF). Net responses were calculated by subtracting the MAIT cell responses to B-LCLs infected with *S*. Typhi from the responses of the controls (uninfected B-LCLs). A ROC AUC curve is a plot of the true positive rates (Sensitivity) in the function of the false positive rates (100-Specificity). Red dotted lines represent the threshold where values above or below the line indicate increases or decreases in performance, respectively. *P* values < 0.05 were considered statistically significant. ROC AUC was generated using absolute MAIT cell numbers per microliter of peripheral blood.

Because the individual analysis of IL-17A yielded good ROC AUC predictive values, and PCA demonstrates a possible significant role for the IFN-γ^−^ TNF-α^−^ IL-17A^+^, IFN-γ^−^TNF-α^+^IL-17A^+^, and IFN-γ^+^TNF-α^+^IL-17A^−^ MAIT cell subsets, we hypothesized that by increasing the number of parameters, it might be possible to increase the power of ROC AUC analysis to discriminate NoTF and TF volunteers based on the production of multiple cytokines. As shown in Fig. 7, the ROC AUC analysis showed a good discriminative ability of the NoTF and TF on 2 polyfunctional MAIT cell subsets: IFN-γ^−^TNF-α^−^IL17A^+^ (AUC = 0.8056; *P* = 0.0394), and IFN-γ^−^TNF-α^+^IL17A^+^ (AUC = 0.8472; *P* = 0.0192). Fair discriminative ability was also found in IFN-γ^+^TNF-α^+^IL17A^−^ (AUC = 0.7922; *P* = 0.0416) MAIT cell subset (Fig. 7). Interestingly, triple-positive IFN-γ^+^TNF-α^+^IL17A^+^, double-positive IFN-γ^+^TNF-α^−^IL17A^+^, and single positive IFN-γ^+^ TNF-α^−^IL17A^−^ MAIT cells were not found to have the discriminative ability (Fig. 7). Thus, the ROC AUC analysis further suggests a key role for IFN-γ^−^TNF-α^−^ IL-17A^+^, IFN-γ^−^ TNF-α^+^IL-17A^+^, and IFN-γ^+^TNF-α^+^ IL-17A^−^ MAIT cell subsets to discriminate between NoTF and TF participant groups. These results also showed that defined cytokine pattern(s) of MAIT cell responses, rather than the average number of selected cytokines expressed, might be associated with disease outcome.

### 3.6. Polyfunctional MAIT cell homing and exhaustion patterns following challenge with S. Typhi

**Fig. 8.**
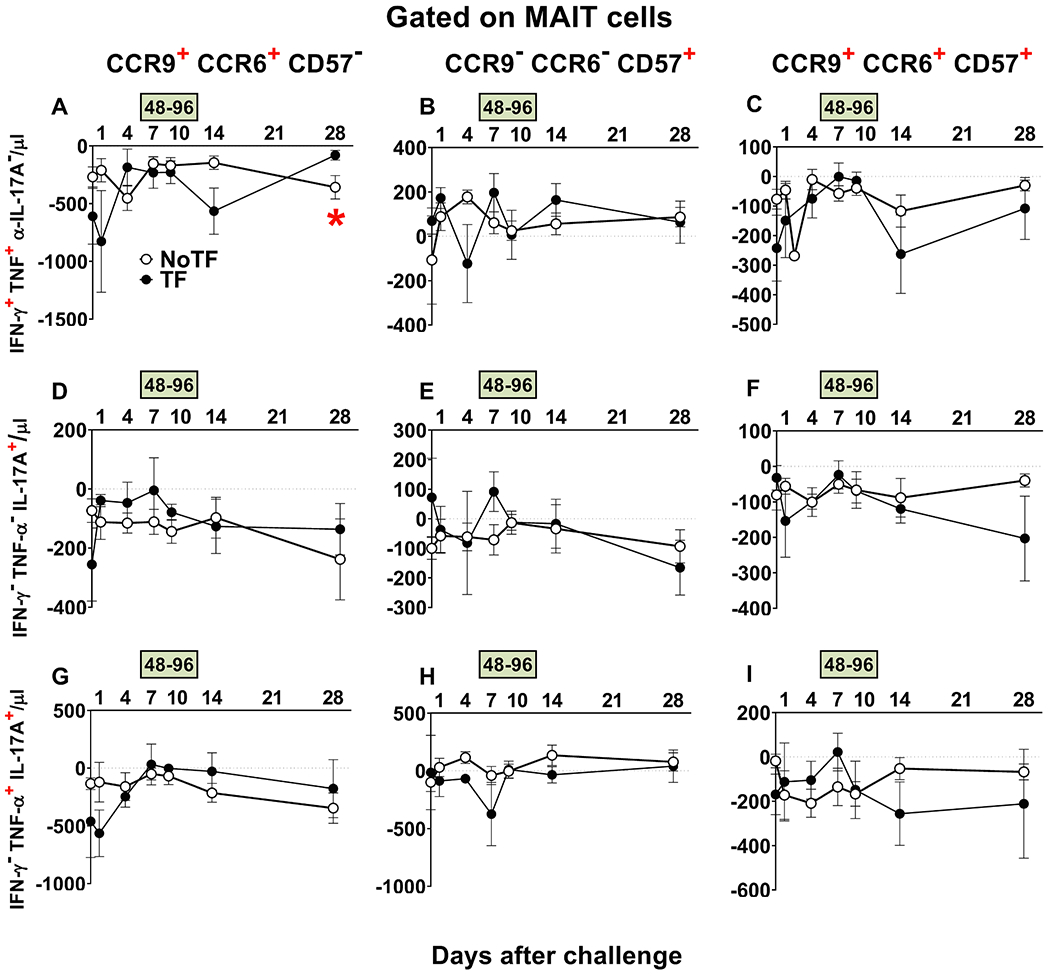
Evaluation of homing and exhaustion markers expressed on monofunctional and polyfunctional MAIT cells over a 28-day post-challenge follow-up period. *Ex vivo* PBMC from participants receiving the *S*. Typhi inoculum were stained, and mono and polyfunctional MAIT cells gated as described in Fig. 3. The curves represent the mean of the net responses, and the bands denote the standard errors of these responses in NoTF (○) and TF (●) participants. Net responses were calculated by subtracting the MAIT cell responses of the controls (uninfected B-LCLs) from those to B-LCLs infected with *S*. Typhi. Only MAIT cell functional signatures suggestive of having an impact on disease outcome based on PCA analysis (Fig. 6 and text) were used to evaluate the concomitant expression of homing (i.e., CCR6 & CCR9) and exhaustion (i.e., CD57) markers. FCOM analysis was employed to study the 7 possible combinations of CCR6 & CCR9 and CD57 expression on MAIT cells. The dashed lines represent the baseline values (day 0). Numbers in the “X” axis represent days after challenge, except for the numbers inside of the green box that represent 48 and 96 hrs after diagnosis of typhoid disease. *, represent significant differences (*P* < 0.05) between the NoTF (○) and TF (●) at the same timepoint. The data represent absolute MAIT cell numbers per microliter of peripheral blood.

We next investigated whether defined homing and exhaustion patterns of polyfunctional MAIT cells could be associated with disease status. We measured the expression of CD57 (a molecule involved in exhaustion), and CCR9 and CCR6 (involved in cell homing to the gut and inflamed tissues, respectively). Previously, we found that during typhoid fever, activated MAIT cells expressing CD57, CCR6, or CCR9 increased in all TF participants [7]. Here, only MAIT cell functional signatures suggestive of discriminating NoTF from TF on ROC AUC analysis were evaluated. FCOM analysis was employed to study the 7 possible combinations of CCR6 & CCR9 and CD57 expression on MAIT cell subsets. We observed that regardless of the disease outcome and MAIT cell subset, positive events occurred only in 3 subsets: CCR9^+^ CCR6^+^ CD57^−^, CCR9^−^ CCR6^−^ CD57^+^, and CCR9^+^ CCR6^+^ CD57^+^ (Fig. 8). It is important to note that when positive events for CCR9 were detected, they were always associated with CCR6 expression. Regarding MAIT cell subsets, we observed that the magnitude of the IFN-γ^+^TNF-α^+^IL-17A^−^ MAIT cell subset co-expressing CCR6 and CCR9, but not CD57 markers was significantly higher at day 28 after the challenge in TF participants, than in NoTF participants (Fig. 8A). When evaluating the curves of NoTF and TF of the IFN-γ^+^TNF-α^+^IL-17A^−^ MAIT cell subset, we observed distinct kinetics that trended to be inverse depending on the time point (Fig. 8A). Thus, the differences between TF and NoTF participants might be due, at least in part, to the delayed increase in the numbers of TF IFN-γ^+^TNF-α^+^IL-17A^−^ MAIT cell subset homing to the inflamed gut, only reaching a higher level than NoTF participants at day 28 after challenge.

## 4. Discussion

Recent observations from our group support the idea that polyfunctional T cells might contribute to effective *S*. Typhi immunity [1–6]. However, the contribution of MAIT cell polyfunctionality to the progression of typhoid fever remains unknown. Whether cytokine secreting MAIT cells can enhance or attenuate the clinical severity of bacterial infections has yet to be elucidated. Here, we showed that CD8 + MAIT cells exhibit distinct cytokine profiles suggestive of diverse functional roles, which are likely to be associated with protection against typhoid fever.

We observed that defined cytokine pattern(s) of MAIT cell responses, rather than the average number of selected cytokines expressed, might be associated with disease outcome. For example, PCA and ROC AUC data suggest that both mono (i.e., IFN-γ^−^ TNF-α^−^ IL-17A^+^) and double (i.e., IFN-γ^−^TNF-α^+^IL-17A^+^ and IFN-γ^+^TNF-α^+^IL-17A^−^) positive MAIT cell subsets might exhibit distinct behavior patterns in NoTF and TF participants. Remarkably, IFN-γ^−^TNF-α^+^IL-17A^+^ and IFN-γ^+^TNF-α^+^IL-17A^−^ MAIT cell phenotypes resemble other bacterial systems in which MAIT cell stimulation involves robust production of IFN-γ and TNF-α [33]. Of note, IFN-γ^+^TNF-α^−^IL-17A^−^ or IFN-γ^+^TNF-α^+^IL-17A^+^ MAIT cells showed no associations with disease outcome. These results support the notion that the pattern(s) of mono and polyfunctional MAIT cell responses, rather than the average number of functionalities expressed by them, is(are) associated with clinical outcomes.

PC analyses also showed that TF MAIT T cell subsets displayed tighter clustering than NoTF MAIT cell subsets. These results suggest that protection against typhoid disease likely requires flexibility in the interplay between MAIT cell subsets resulting in the activation of multiple signaling pathways. TF MAIT cell responses where subsets tend to change in unison might be deleterious for disease outcome. They might activate a limited number of signaling pathways which might not be optimal for protection from disease. Indeed, recently Toubal *et al*. showed that crosstalk between MAIT cells and M1 macrophages promotes MAIT cell inflammatory function [34]. MAIT cells cultured in the presence of M1 macrophages expressed higher levels of IL-17A, and TNF-α, as compared with cultures with M0 or M2 macrophages [34]. Supporting these findings, we found that the crosstalk among leukocytes-lymphocytes, macrophages, and PMN- is cytokine/chemokine-dependent and might play a pivotal role in orchestrating the functional efficiency of innate cells against bacterial infection [35].

Our previous *ex-vivo* studies have shown that MAIT cells from TF participants acquire different homing and exhaustion patterns than those observed in NoTF participants [7]. Here we extended these studies by showing a delayed increase in the numbers of TF IFN-γ^+^TNF-α^+^IL-17A^−^ MAIT cell subset homing to the inflamed gut (*i.e.*, CCR6^+^ CCR9^+^), only reaching a higher level than NoTF participants 28 days after the challenge. We also observed an increase in the percentage of exhausted IFN-γ^+^TNF-α^+^IL-17A^+^MAIT cell subset during the period of typhoid disease. These results are consistent with our recent work showing an association between the HLA-G expression on B cells and the regulation of IFN-γ production by MAIT cells, resulting in their loss [23]. Because CCR6 & CCR9 markers are involved in homing to the inflamed gut, and in this manuscript we studied MAIT cell populations in circulation, it is possible that some of the MAIT cell subsets might have already migrated to the gut and other tissues and are no longer in circulation to be measured.

The investigators are well aware that the relatively small number of participants available for the studies presented in this manuscript is an overall weakness. However, it is important to note that this issue was mainly due to the limitations inherent in using human samples, especially after challenges with wild-type organisms. While the limited number of samples might have affected the study if the null hypothesis was not rejected, this was not the case when we observed statistically significant differences between participants allocated to NoTF and TF groups.

Although this study did not intend to examine differences between NoTF and TF groups according to sex, it is interesting to note that none of the female participants developed TF (S1 Table). These findings prompted us to review the demographic data from the original 41 participants. By using Maryland’s definition of typhoid fever, we found a significantly higher proportion of males among TF in comparison to the NoTF group (*P* > 0.0091, two-sided Chi-square, or *P* > 0.0087, two-sided Fisher’s Exact test). Although, the biological explanations for these findings are unclear and beyond the scope of this already lengthy manuscript, these findings corroborated previous studies showing gender differences in the frequency of MAIT cells [36,37]. For example, it has been estimated that the annual decline in circulating MAIT cell level is 3.2% in men and 1.8% in women [37]. These results also agree with previous studies showing that generally, males exhibit lower immune responses to vaccination than females, including to a Vi conjugate typhoid vaccine [38–40].

In sum, by analyzing the profile of cytokine production by MAIT cell subsets, it was possible to identify cytokine patterns that may serve as predictive biomarkers during natural infection and vaccination. In addition, MAIT cells are prevalent at mucosas (*i.e.*, key sites of bacteria entry), devoid of alloreactivity, and able to expand *in vitro* [41,42]. Thus, manipulating their properties to express defined cytokine signature(s) during *in vitro* expansion or following administration of immunomodulators might help fight bacterial infections such as salmonellosis.

## Supplementary Material

Supplemental Figures

Figure Legend for Supplementals

## Abbreviations:

MAIT cells: Mucosal-Associated Invariant T cells
*S*. Typhi: *Salmonella enterica* serovar Typhi
TF: participants who had fever ≥38°C followed by *S*. Typhi isolation from blood
NoTF: participants who did not meet the typhoid fever definition
