# Supplementary material for: Mucosal-Associated Invariant T cells exhibit distinct functional signatures associated with protection against typhoid fever
Source: Cell Immunol. Author manuscript; Available in PMC 2022 Aug 16. (PMC9377420; doi:10.1016/j.cellimm.2022.104572)
Supplement: Supplemental Figures [file NIHMS1822276-supplement-Supplemental_Figures.pdf]

Mucosal-Associated Invariant T cells exhibit distinct functional signatures associated with protection against typhoid fever

Rosângela Salerno-Goncalves, Stephanie Fresnay, Laurence Magder, Thomas C. Darton, Claire S. Waddington, Christoph J. Blohmke, Brian Angus, Myron M. Levine, Andrew J. Pollard, and Marcelo B. Sztein

Supplementals

S1 Table. Demographics of the participants included in this study

	NoTF* (N=13)	TF** (N=7)
**Age**		
Mean (SD)	29 (± 7)	30 (± 10)
Range	19-43	20-46
**Gender**		
Female	6 (46)	0 (0)
Male	7 (54)	7 (100)
***S*. Typhi challenge dose**		
Mean	1.54×10^4	1.18×10^4
Range	$0.07\text{-}2.7 \times 10^4$	$0.1\text{-}2.7 \times 10^4$
**Time to Clinical Diagnosis, day**		
Mean	-	7
Range	-	6-9
**Total Lymphocyte count, x $10^3/\mu l$, mean (SD)**		
Day 0	2.05 (± 0.41)	2.53 (± 1.41)
Day 1-4	2.08 (± 0.65)	2.23 (± 1.17)
Day 7-10 (48-96 hs)†	1.70 (± 0.58)	1.03 (± 0.42)
Day 14-28	1.97 (± 0.59)	2.22 (± 1.04)
**Symptoms**		
Fever > 38°C followed by *S*. Typhi isolation from blood culture	-	7
Neither fever nor bacteremia	9	-
Bacteremia only	3	-
Fever only	1	-

*, NoTF, volunteers who did not meet the clinical typhoid fever definition; **, TF, volunteers with typhoid fever diagnosis;
† , *t*-test NoTF vs. TF, *P* =0.0002

S1 Figure

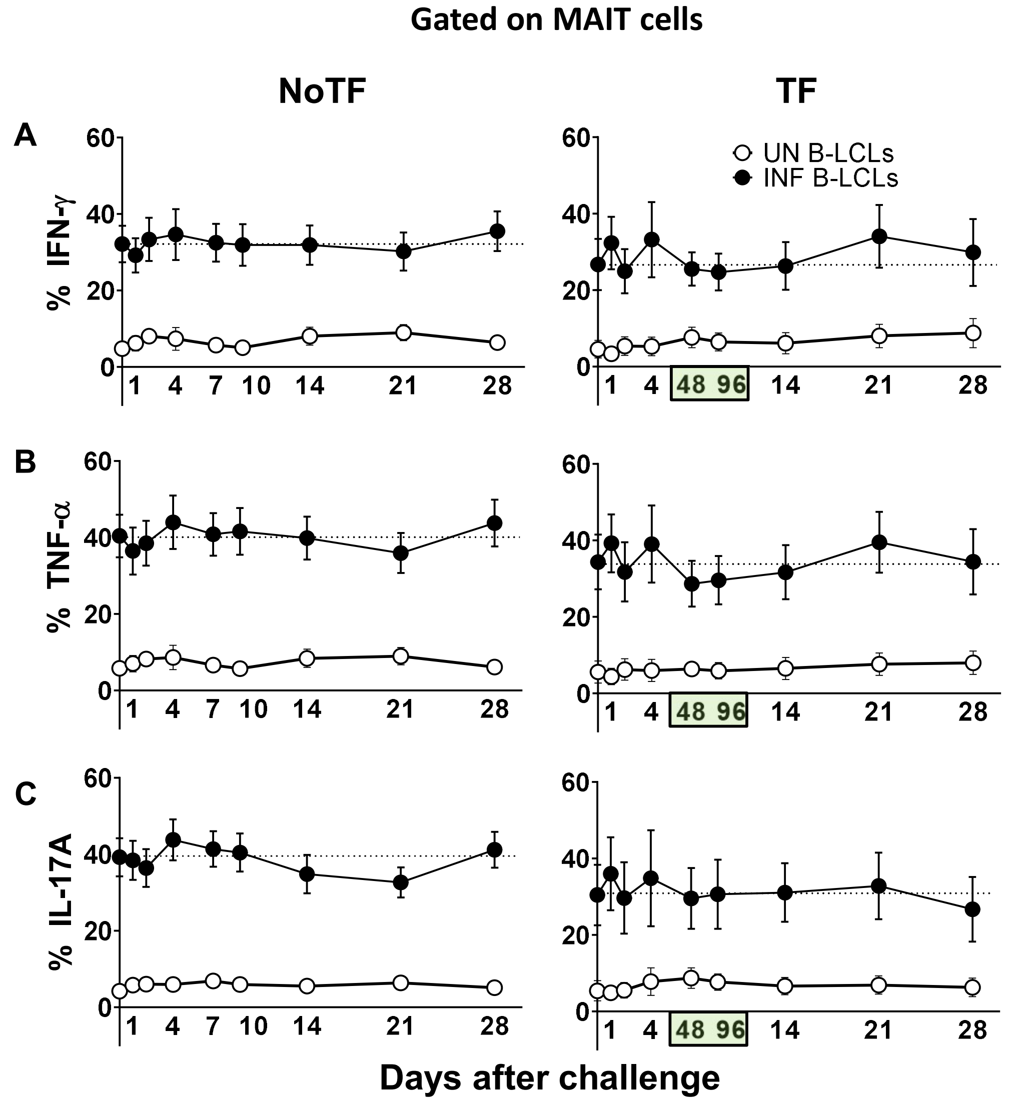
Gated on MAIT cells
NoTF
TF
A
B
C
UN B-LCLs
INF B-LCLs
% IFN-γ
% TNF-α
% IL-17A
60
40
20
0
1
4
7
10
14
21
28
48 96
Days after challenge

S2 Figure

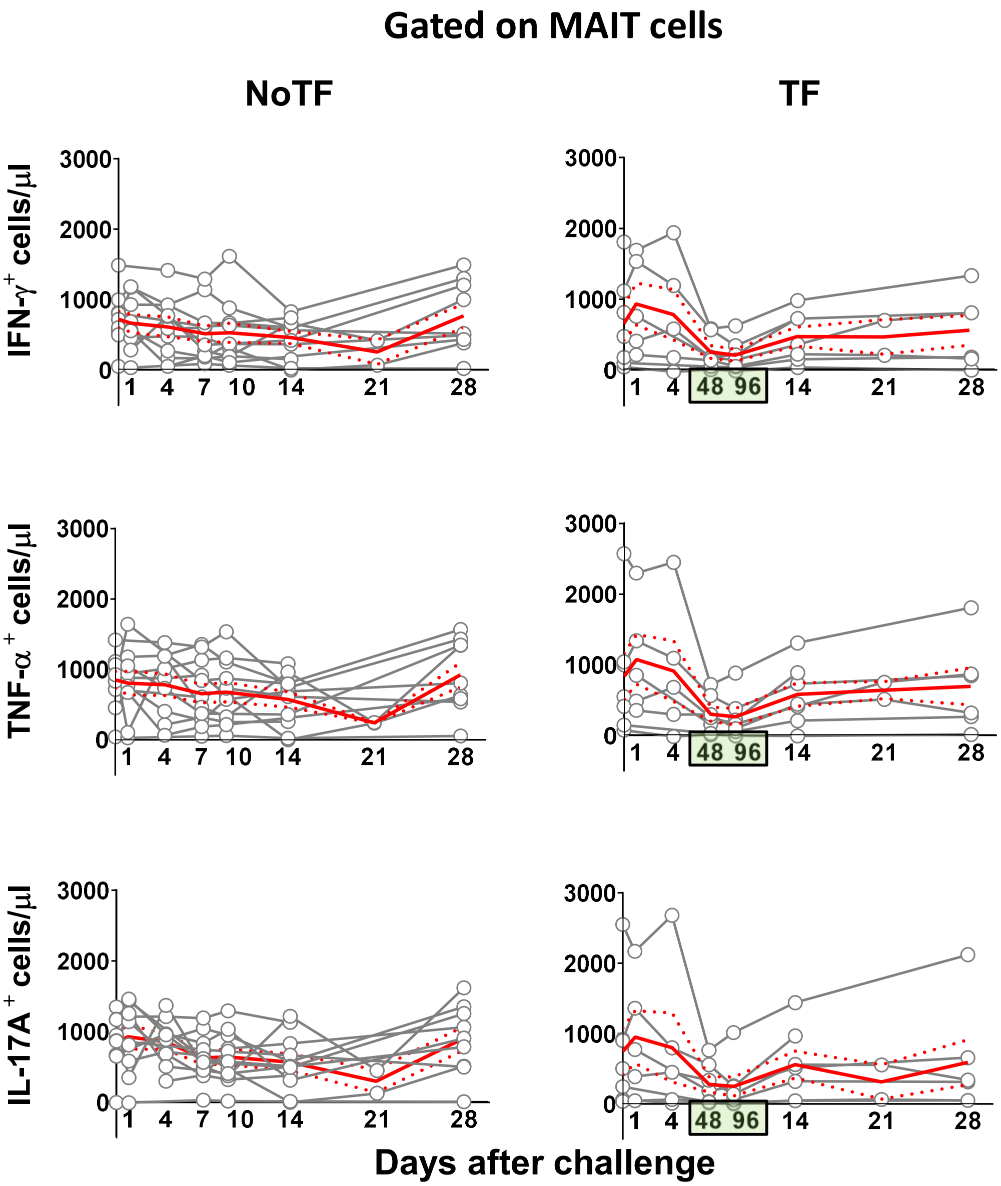
Gated on MAIT cells
NoTF
TF
IFN-γ+ cells/µl
TNF-α + cells/µl
IL-17A + cells/µl
3000
2000
1000
0
1 4 7 10 14 21 28
1 4 48 96 14 21 28
Days after challenge

S3 Figure

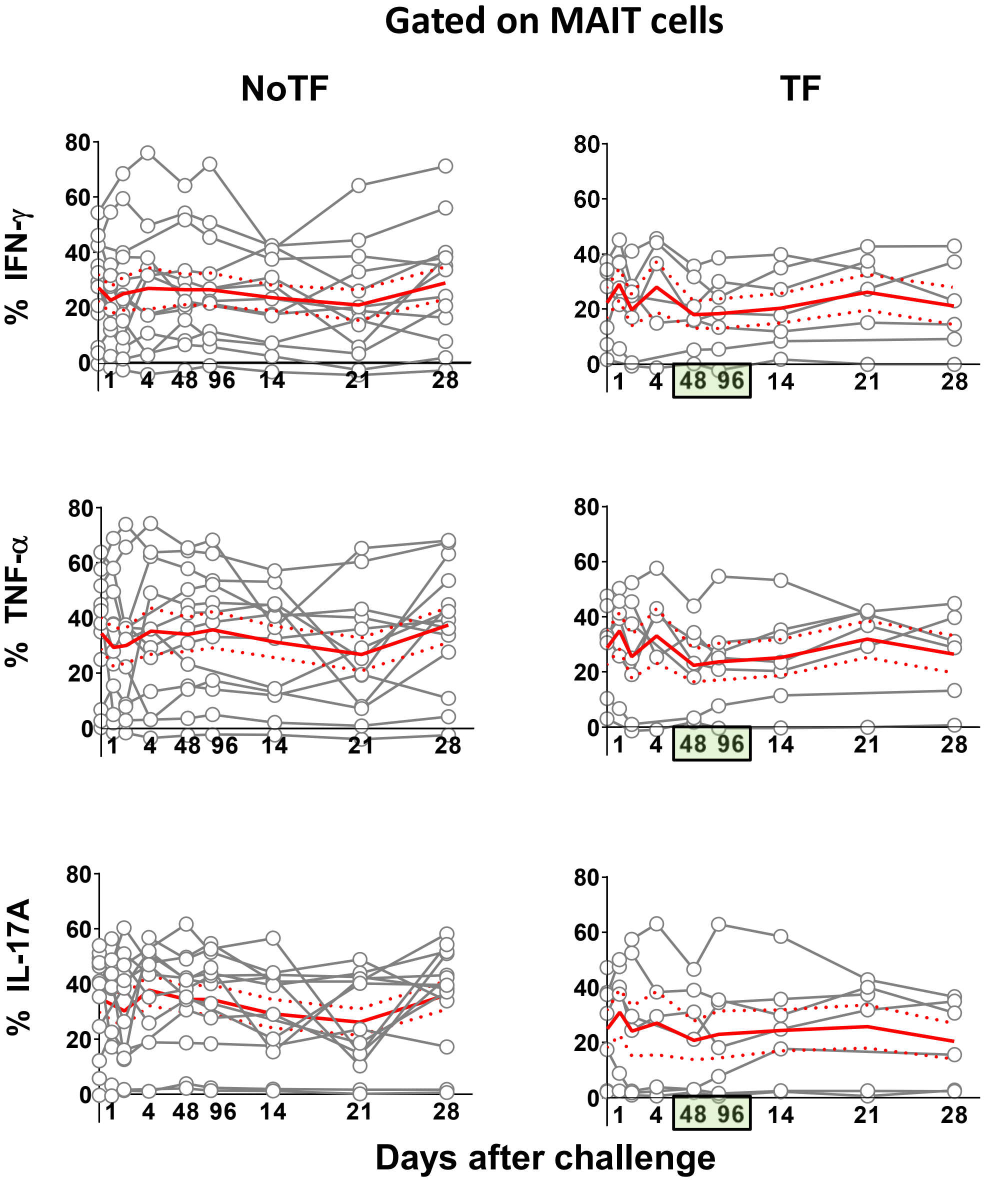

Gated on MAIT cells
NoTF
TF
% IFN-γ
% TNF-α
% IL-17A
80
60
40
20
0
1
4
48
96
14
21
28
Days after challenge

S4 Figure

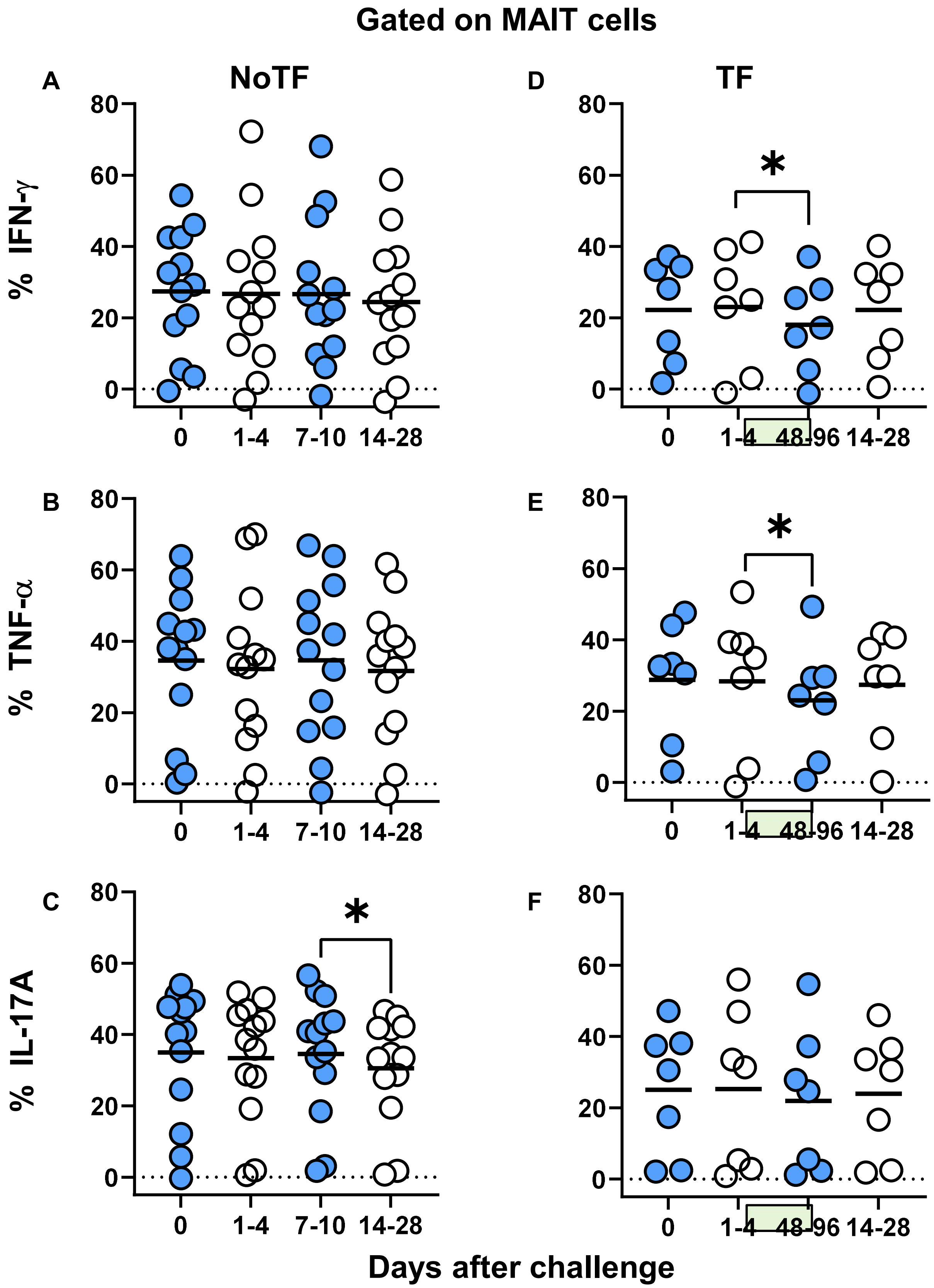

Gated on MAIT cells
A
NoTF
D
TF
B
E
C
F
% IFN-γ
% TNF-α
% IL-17A
80
60
40
20
0
*
0
1-4
7-10
14-28
48-96
Days after challenge

S5 Figure

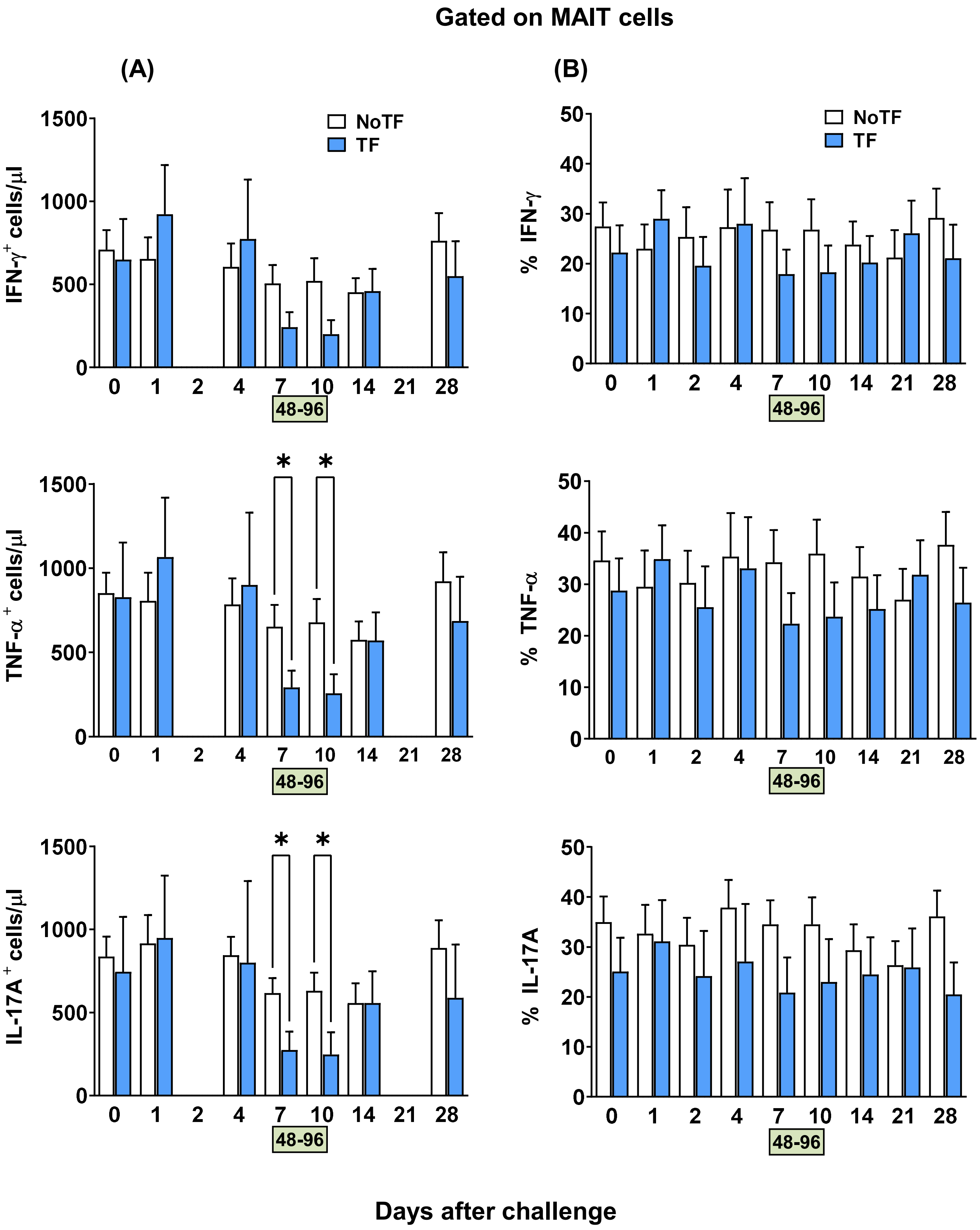
Gated on MAIT cells
(A)
(B)
NoTF
TF
IFN-γ+ cells/μl
% IFN-γ
TNF-α + cells/μl
% TNF-α
IL-17A + cells/μl
% IL-17A
0
500
1000
1500
10
20
30
40
50
0
1
2
4
7
10
14
21
28
48-96
*
Days after challenge

S6 Figure

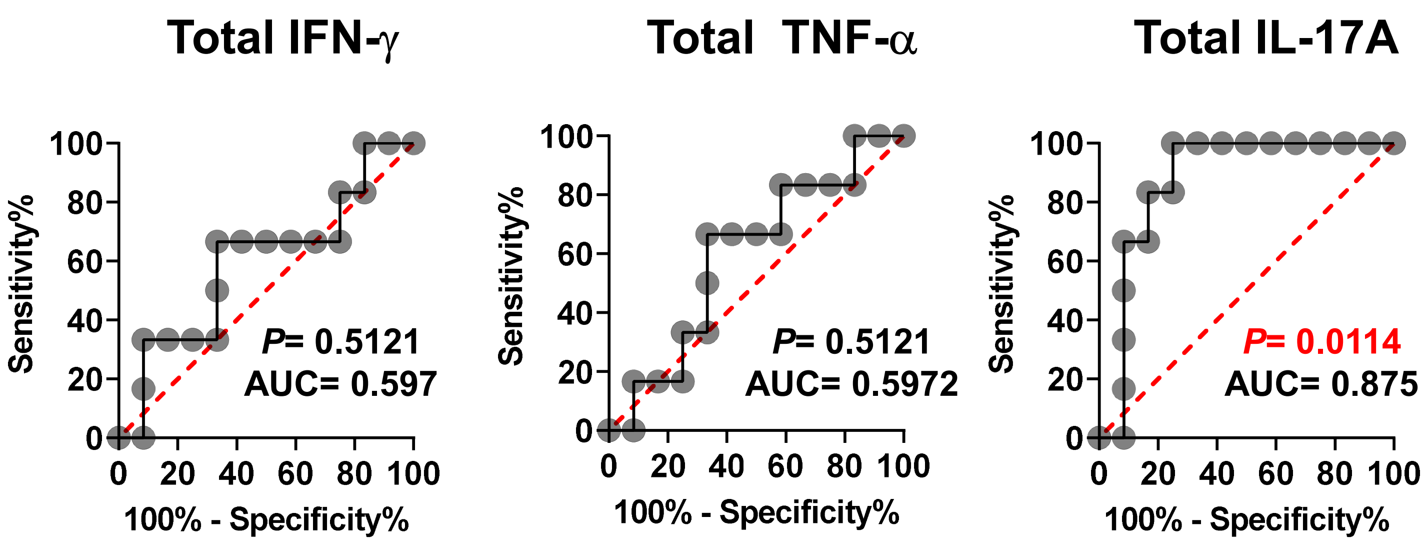

Total IFN-γ
Sensitivity%
100
80
60
40
20
0
0 20 40 60 80 100
100% - Specificity%
P= 0.5121
AUC= 0.597
Total TNF-α
Sensitivity%
100
80
60
40
20
0
0 20 40 60 80 100
100% - Specificity%
P= 0.5121
AUC= 0.5972
Total IL-17A
Sensitivity%
100
80
60
40
20
0
0 20 40 60 80 100
100% - Specificity%
P= 0.0114
AUC= 0.875

S7 Figure

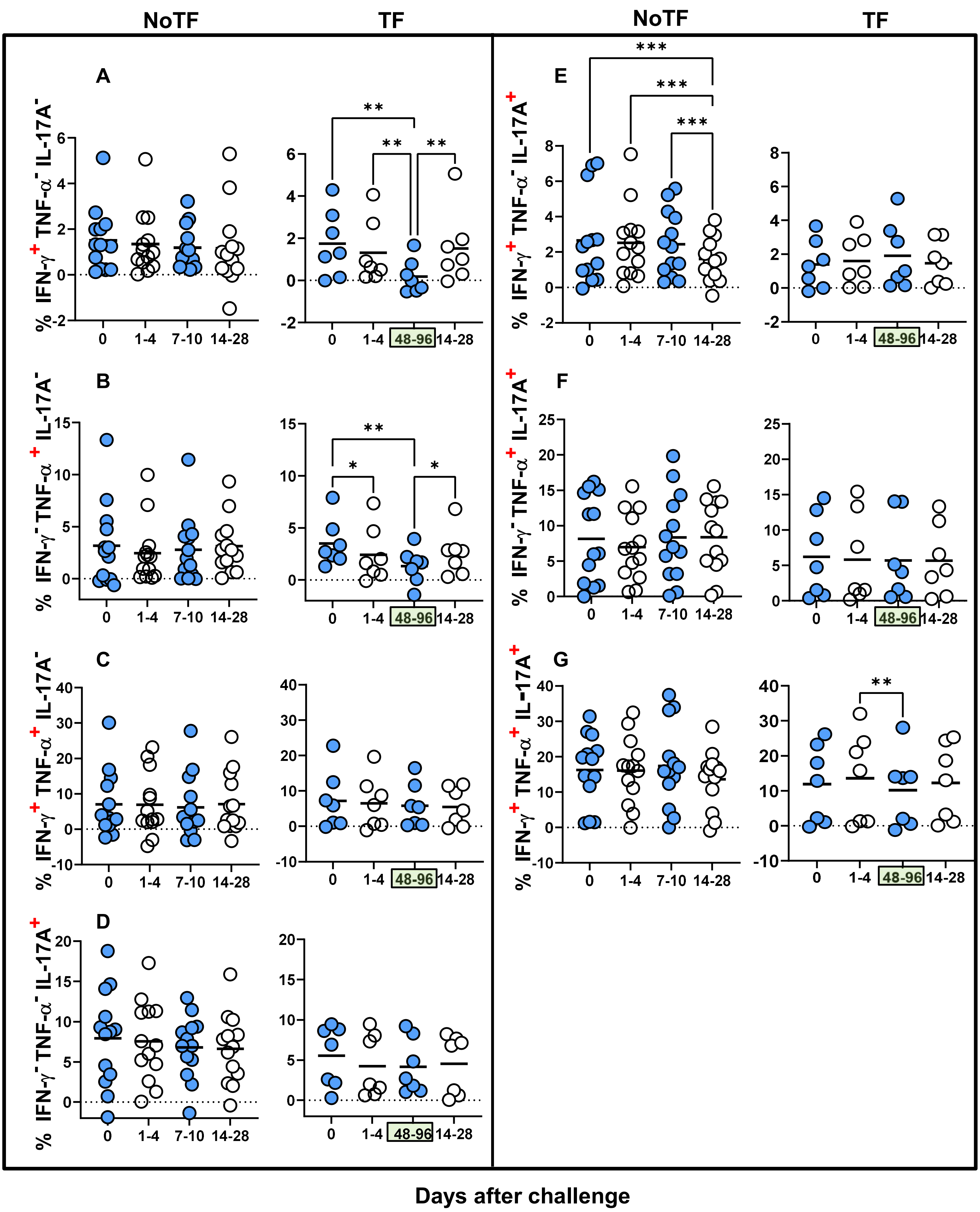

NoTF
TF
NoTF
TF
A
B
C
D
E
F
G
% IFN-γ+ TNF-α- IL-17A-
% IFN-γ- TNF-α+ IL-17A-
% IFN-γ+ TNF-α+ IL-17A-
% IFN-γ- TNF-α- IL-17A+
% IFN-γ+ TNF-α- IL-17A+
% IFN-γ- TNF-α+ IL-17A+
% IFN-γ+ TNF-α+ IL-17A+
0
1-4
7-10
14-28
48-96
**
***
*
Days after challenge

S8 Figure

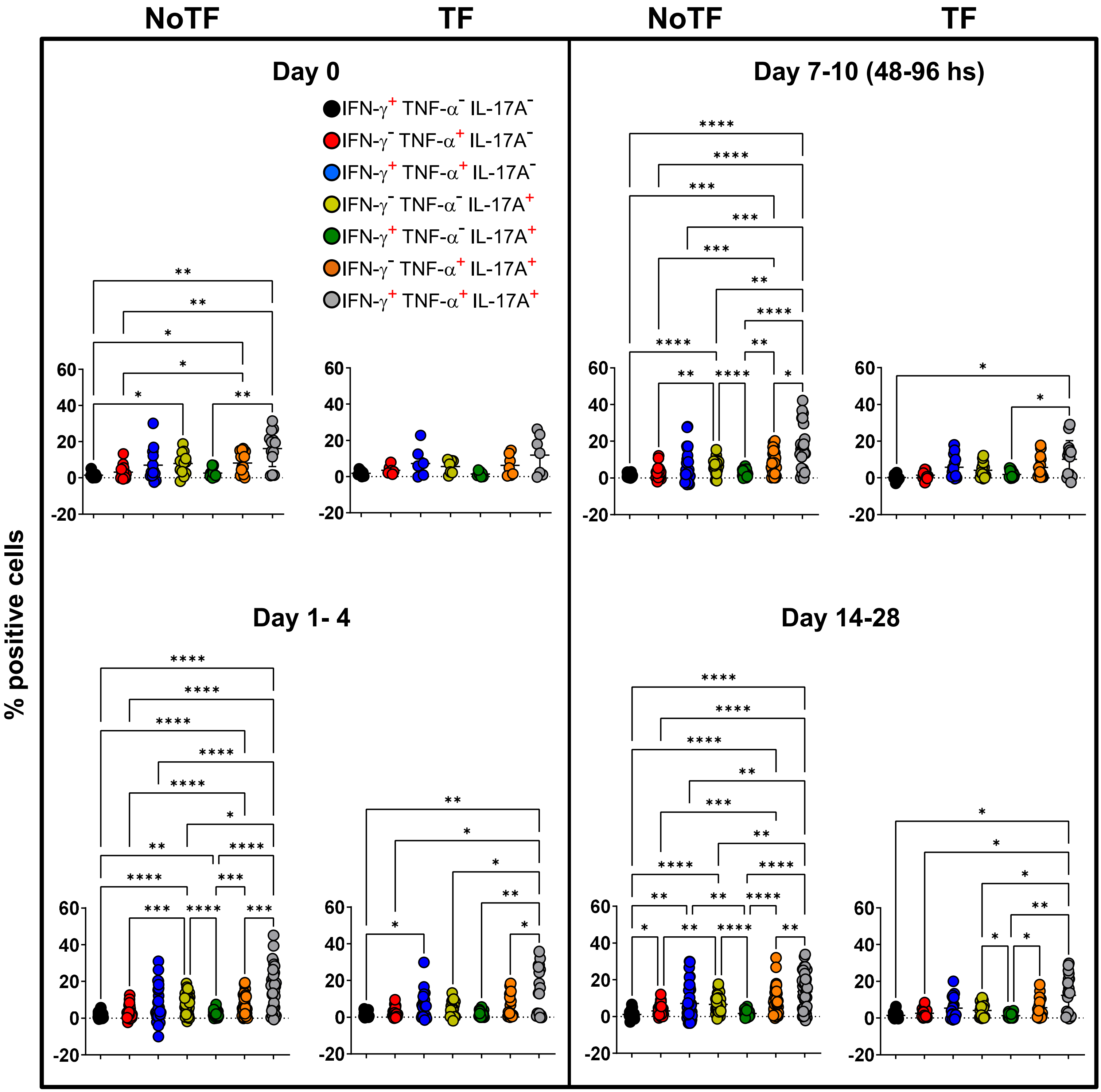
NoTF
TF
NoTF
TF
Day 0
Day 7-10 (48-96 hs)
Day 1- 4
Day 14-28
IFN-γ+ TNF-α- IL-17A-
IFN-γ- TNF-α+ IL-17A-
IFN-γ+ TNF-α+ IL-17A-
IFN-γ- TNF-α- IL-17A+
IFN-γ+ TNF-α- IL-17A+
IFN-γ- TNF-α+ IL-17A+
IFN-γ+ TNF-α+ IL-17A+
% positive cells

S9 Figure

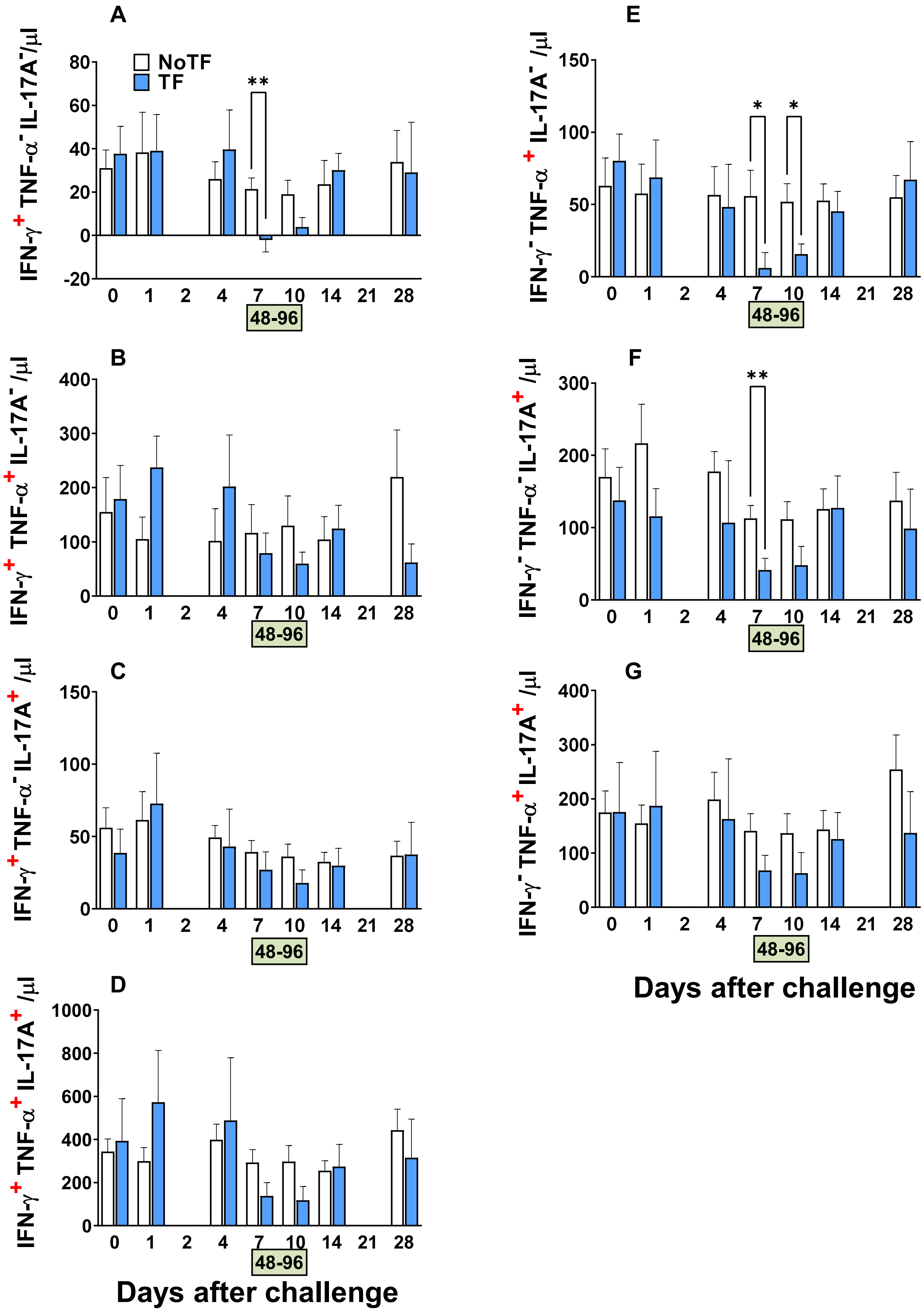

A
E
B
F
C
G
D
NoTF
TF
IFN-γ+ TNF-α- IL-17A-/μl
IFN-γ- TNF-α+ IL-17A- /μl
IFN-γ+ TNF-α+ IL-17A- /μl
IFN-γ- TNF-α- IL-17A+ /μl
IFN-γ+ TNF-α- IL-17A+ /μl
IFN-γ- TNF-α+ IL-17A+ /μl
IFN-γ+ TNF-α+ IL-17A+ /μl
0 1 2 4 7 10 14 21 28
48-96
Days after challenge

S10 Figure

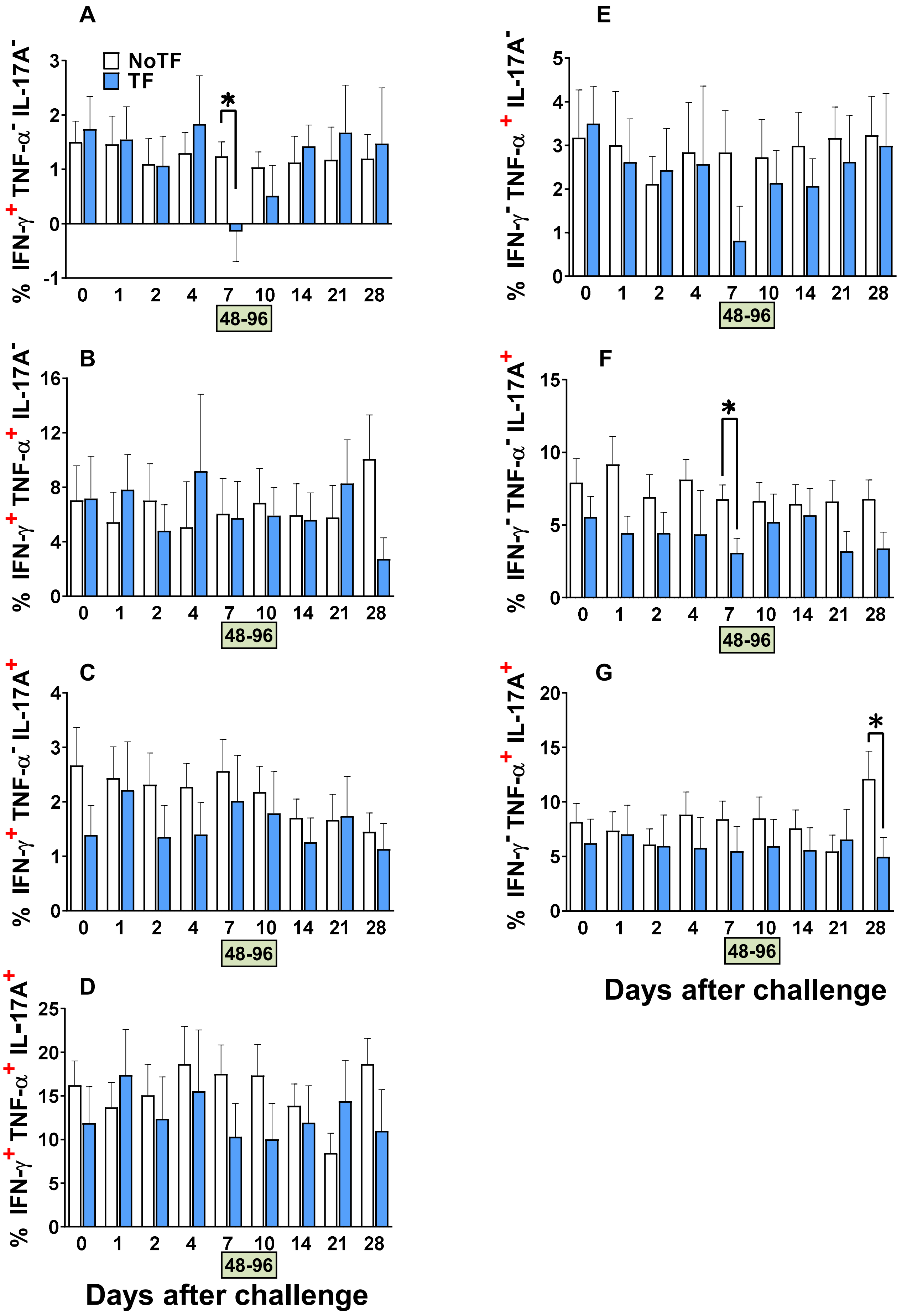

A
B
C
D
E
F
G
NoTF
TF
% IFN-γ+ TNF-α- IL-17A-
% IFN-γ+ TNF-α+ IL-17A-
% IFN-γ+ TNF-α- IL-17A+
% IFN-γ+ TNF-α+ IL-17A+
% IFN-γ- TNF-α+ IL-17A-
% IFN-γ- TNF-α- IL-17A+
% IFN-γ- TNF-α+ IL-17A+
0
1
2
4
7
10
14
21
28
48-96
*
Days after challenge
