# Supplementary material for: Mucosal-Associated Invariant T cells exhibit distinct functional signatures associated with protection against typhoid fever
Source: Cell Immunol. 2022 Aug;378:None. doi: 10.1016/j.cellimm.2022.104572 (PMC9377420; doi:10.1016/j.cellimm.2022.104572)
Supplement: Supplementary file 1 [file mmc2.pdf]

Supplementary Figures

S1 Figure. **Kinetics of cytokine-producing MAIT cells from participants over a 28-day post-challenge follow-up period.** *Ex vivo* PBMC from participants receiving a *S*. Typhi inoculum were stained with YEVID, followed by surface staining with mAbs to CD3, CD4, CD8, CD14, CD19, CD161, and TCRα 7.2. After fixation and permeabilization, cells were intracellularly stained with monoclonal antibodies to CD69, as well as to IFN-γ, TNF-α, and IL-17A cytokines and analyzed by flow cytometry. For the analysis, following the elimination of doublets and other debris, a "dump" channel was used to eliminate dead cells (YEVID^{+}) as well as macrophages (CD14^{+}), and B cells (CD19^{+}) from the analyses. This was followed by additional gating on CD3, CD4, and CD8, as well as CD161 versus TCRα 7.2 to analyze MAIT cells, and afterward on CD69, IFN-γ, TNF-α, and IL-17A to evaluate cytokine secretion. Kinetics of the production of (**A**) IFN-γ, (**B**) TNF-α, and (**C**) IL-17A by MAIT cells following exposure to (●) autologous B-LCLs infected with *S*. Typhi (INF B-LCLs), or controls (○) (UN, uninfected B-LCLs). The curves represent the mean, and the bands denote the standard errors of the results from the 13 participants who did not meet the clinical typhoid fever definition (NoTF), and the 7 who did (TF). The dashed lines represent the baseline values (day 0). Numbers in the "X" axis represent days after challenge, except for the numbers inside of the green box that represent 48 and 96 hrs after diagnosis of typhoid disease. Data are presented as percentages of MAIT cells.

S2 Figure. **Kinetics of cytokine-producing MAIT cells from individual participants over a 28-day post-challenge follow-up period.** *Ex vivo* PBMC were stained and MAIT cells gated as described in **S1 Figure**. Grey lines represent individual net responses in 13 participants who did not meet the clinical typhoid fever definition (NoTF), and 7 who did (TF). Net responses were

calculated by subtracting the MAIT cell responses of the controls (uninfected B-LCLs) from those observed to autologous B-LCLs infected with *S*. Typhi. The red lines represent the mean of the results from the 13 NoTF and 7 TF participants. Numbers in the "X" axis represent days after challenge, except for the numbers inside of the green box that represent 48 and 96 hrs after diagnosis of typhoid disease. Data are presented as absolute MAIT cell numbers per microliter of peripheral blood.

S3 Figure. **Kinetics of cytokine-producing MAIT cells from individual participants over a 28-day post-challenge follow-up period.** *Ex vivo* PBMC were stained and MAIT cells gated as described in **S1 Figure**. Grey lines represent individual net responses in 13 participants who did not meet the clinical typhoid fever definition (NoTF), and 7 who did (TF). Net responses were calculated by subtracting the MAIT cell responses of the controls (uninfected B-LCLs) from those observed to B-LCLs infected with *S*. Typhi. The red lines represent the mean of the results from the 13 NoTF and 7 TF participants. Numbers in the "X" axis represent days after challenge, except for the numbers inside of the green box that represent 48 and 96 hrs after diagnosis of typhoid disease. Data are presented as percentages of MAIT cells.

S4 Figure. **Comparison of cytokine-producing MAIT cell kinetics over the 28-day post-challenge follow-up period.** *Ex vivo* PBMC from participants receiving the *S*. Typhi inoculum were stained and analyzed as described in the legend to **Figure S1**. Levels of MAIT cells expressing IFN-γ (**A** & **D**), TNF-α (**B** & **E**), and IL-17A (**C** & **F**), were evaluated in NoTF and TF participants. Data were grouped by time frames as follows: day 0, days 1-4, days 7-10 (for NoTF), or 48-96 hours for TF participants and days 14-28. For grouped timepoints, each dot represents

the mean value of the different timepoints for an individual participant. NoTF, participants who did not meet the clinical typhoid fever definition; TF, participants who did meet the clinical typhoid fever definition. *, represent significant differences (*P*<0.05). Data are presented as percentages of MAIT cells.

S5 Figure. Comparison of the kinetics of cytokine-secreting MAIT cells among NoTF and TF participants over the 28-day post-challenge follow-up period. *Ex vivo* PBMC were stained and MAIT cells gated as described in **Figure 1**. The bars represent the mean, and the bands denote the standard errors of net MAIT cell responses. Data are representative of the net responses in 13 participants who did not meet the clinical typhoid fever definition (□, NoTF), and 7 who did (■, TF): (**A**) absolute numbers per microliter, and (**B**) percentages of MAIT cells. Net responses were calculated by subtracting the MAIT cell responses of the controls (uninfected B-LCLs) from those observed to B-LCLs infected with *S*. Typhi. *, represent significant differences (*P*<0.05). **Note**: Since the absolute lymphocyte values in peripheral blood were missing for all participants on day 2, and for most participants on day 21, these two time points were eliminated from the analysis.

S6 Figure. Ability of ROC AUC analysis of cytokine secreting MAIT cells to discriminate clinical outcome in volunteers receiving low-dose vs high-dose inocula. *Ex vivo* PBMC were stained, and IFN-γ, TNF-α, and IL-17A secreting MAIT cells gated as described in **Figure S1**. ROC curve logistic regressions were based on net MAIT cell responses of volunteers who did not meet the definition of typhoid fever (NoTF), and those who did (TF). Net responses were calculated by subtracting the MAIT cell responses to autologous B-LCLs infected with *S*. Typhi from the responses of the controls (uninfected B-LCLs). ROC AUC curves are plots of the true

positive rates (Sensitivity) in function of the false positive rates (100-Specificity). The red dotted line represents the threshold where values above or below indicate increases or decreases in performance, respectively. *P* values <0.05 were considered statistically significant. ROC AUC was generated using absolute MAIT cell numbers per microliter of peripheral blood.

S7 Figure. Comparison of mono and polyfunctional MAIT cell kinetics over a 28-day post-challenge follow-up period. *Ex vivo* PBMC from participants receiving the *S.* Typhi inoculum were stained and MAIT cells gated as described in **Figure S1**. FCOM, an analysis tool that automatically reduces multiparameter data to a series of multiple event acquisition gates, one for every possible sub-phenotype, was employed to study MAIT cell polyfunctionality. Based on the pre-defined positive staining regions for cytokines, FCOM calculated 7 possible phenotypes as displayed in the figure legend. Data are representative of 13 participants who did not meet the clinical typhoid fever definition (NoTF), and 7 who did (TF). Data are the net responses calculated by subtracting the MAIT cell responses of the controls (uninfected B-LCLs) from those observed to B-LCLs infected with *S.* Typhi, and grouped by time frames as follows: day 0, days 1-4, days 7-10 (for NoTF, or 48-96 hours for TF participants), and days 14-28. For grouped timepoints, each dot represents the mean value of the different time points for an individual participant. Asterisks describe the levels of statistical significance as: ***, extremely significant (*P*-value between 0.0001 to 0.001); **, very significant (*P*-value between 0.001 to 0.01); *, significant (*P*-value between 0.01 to 0.049); *P* values <0.05 were considered statistically significant. Data are presented as percentages of MAIT cells expressing a particular cytokine combination.

S8 Figure. Comparison among monofunctional and polyfunctional MAIT cells over a 28-day

post-challenge follow-up period. *Ex vivo* PBMC were stained and MAIT cells gated as described in **Figure S1**. FCOM, an analysis tool that automatically reduces multiparameter data to a series of multiple event acquisition gates, one for every possible sub-phenotype, was employed to study MAIT cell polyfunctionality. Based on the pre-defined positive staining regions for cytokines, FCOM calculated 7 possible phenotypes as displayed in the figure legend. Data are representative of 13 participants who did not meet the clinical typhoid fever definition (NoTF), and 7 who did (TF). Data are the net responses calculated by subtracting the MAIT cell responses of the controls (uninfected B-LCLs) from those observed to B-LCLs infected with *S*. Typhi, and grouped by time frames as follows: day 0, days 1-4, days 7-10 (for NoTF, or 48-96 hours for TF participants) and days 14-28. Asterisks describe the levels of statistical significance as: ****, extremely significant (*P* <0.0001); ***, extremely significant (*P*-value between 0.0001 to 0.001); **, very significant (*P*-value between 0.001 to 0.01); *, significant (*P*-value between 0.01 to 0.049); *P* values < 0.05 were considered statistically significant. Data are presented as percentages of MAIT cells expressing a particular cytokine combination.

S9 Figure. Comparison of the kinetics of monofunctional and polyfunctional MAIT cells among NoTF and TF participants over a 28-day post-challenge follow-up period. *Ex vivo* PBMC were stained and MAIT cells gated as described in **Figure 1**. The bars represent the mean, and the bands denote the standard errors of net MAIT cell responses. Data are representative of the net responses in 13 participants who did not meet the clinical typhoid fever definition (□ , NoTF), and 7 who did (■ , TF). Net responses were calculated by subtracting the MAIT cell responses of the controls (uninfected B-LCLs) from those observed to B-LCLs infected with *S*. Typhi. *, represent significant differences (*P*<0.05) between NoTF and TF at the same timepoint.

The data represent absolute MAIT cell numbers per microliter of peripheral blood expressing a particular cytokine combination. **Note**: Since the absolute lymphocytes values in peripheral blood were missing for all participants on day 2, and for most participants on day 21, these two time points were eliminated from the analysis.

S10 Figure. Comparison of the kinetics of monofunctional and polyfunctional MAIT cells among NoTF and TF participants over a 28-day post-challenge follow-up period. *Ex vivo* PBMC were stained and MAIT cells gated as described in **Figure 1**. The bars represent the mean, and the error bars denote the standard errors of net MAIT cell responses. Data are representative of the net responses in 13 participants who did not meet the clinical typhoid fever definition (□, NoTF), and 7 who did (■, TF). Net responses were calculated by subtracting the MAIT cell responses of the controls (uninfected B-LCLs) from those observed to B-LCLs infected with *S*. Typhi. *, represent significant differences (P<0.05) between NoTF and TF at the same timepoint. Data are presented as percentages of MAIT cells expressing a particular cytokine combination.
